# From mechanism to therapeutics: targeting the mitogen-activated protein kinase 1 (MAPK1)/extracellular signal-regulated kinase 2 (ERK2) pathway in renal fibrosis

**DOI:** 10.7717/peerj.21529

**Published:** 2026-07-21

**Authors:** Monan Cao, Jun Fan, Lanyu Jia, Yefan Pu, Bohua Chen, Sihan Zhang, Jiawang Wang

**Affiliations:** 1 Tianjin University of Traditional Chinese Medicine, Tianjin, China; 2 Tianjin Academy of Traditional Chinese Medicine Affiliated Hospital, Tianjin, Hongqiao District, China

**Keywords:** MAPK1/ERK2 signaling pathway, Renal fibrosis, Epithelial-mesenchymal transition, Signal transduction, Therapeutic target, Kinase inhibitor, Cell proliferation and differentiation, Kidney disease, Pharmacological intervention

## Abstract

Renal fibrosis represents a crucial pathological event in the progression of chronic kidney disease (CKD) to end-stage renal disease (ESRD), with currently limited therapeutic options. As a key regulator of cell proliferation and differentiation, the mitogen-activated protein kinase 1 (MAPK1)/extracellular signal-regulated kinase 2 (ERK2) signaling pathway plays a central role in the development of renal fibrosis. This review aims to comprehensively elucidate the regulatory network of the MAPK1/ERK2 pathway, summarize recent advances in targeted therapies against renal fibrosis, and provide directions for developing novel anti-fibrotic drugs. Special attention is given to bioactive compounds, including both chemically synthesized drugs and natural products. Through cextensive literature searches of databases including PubMed and Web of Science, this review discusses how the MAPK1/ERK2 pathway responds to upstream signals to promote myofibroblast activation, inflammation, and aberrant extracellular matrix (ECM) deposition *via* downstream effectors. The review highlights that these compounds demonstrate notable antifibrotic efficacy in preclinical models, primarily by directly or indirectly inhibiting MAPK1/ERK2 phosphorylation. Furthermore, we emphasize that these chemically defined compounds represent promising molecular probes and therapeutic candidates. In conclusion, targeting the MAPK1/ERK2 pathway remains strategically important. Given the current absence of approved anti-fibrotic drugs, in-depth exploration of specific molecular targets within this pathway holds considerable translational potential and may fill a critical gap in this field. This narrative review synthesizes current knowledge on the MAPK1/ERK2 pathway in renal fibrosis, highlighting its regulatory mechanisms and therapeutic promise.

## Introduction

Chronic kidney disease (CKD) is characterized by a gradual deterioration of renal function resulting from diverse etiological factors. Diabetes and hypertension remain the two most common causes, both of which contribute to chronic damage to the renal filtration units (Cockwell & Fisher, 2020). Renal fibrosis represents a key pathological mechanism underlying CKD progression, characterized by excessive fibroblast activation and extracellular matrix (ECM) deposition, ultimately leading to the destruction of functional renal units. Microscopically, this phenomenon manifests as prominent glomerular sclerosis and tubular atrophy, progressively resulting in organ dysfunction (Rayego-Mateos & Valdivielso, 2020). The eventual development of end-stage renal disease (ESRD) due to progressive organ failure explains why CKD now poses an increasingly heavy burden on global public health systems. According to recent epidemiological data, the global prevalence of CKD was 14.2% in 2023, while in China, the prevalence was approximately 10.8% (GBD 2023 Chronic Kidney Disease Collaborators, 2025; Zhang et al., 2012). Both figures have shown continuous increases in recent years (Guo et al., 2025a; Moeller et al., 2021). Since 1990, the number of patients with ESRD has risen dramatically, requiring renal replacement therapies, including dialysis or transplantation (Cockwell & Fisher, 2020). Consequently, CKD significantly contributes to global mortality and imposes substantial financial burdens across society. Given the progressive and currently incurable nature of CKD, strategies aimed at delaying disease progression and managing associated complications have been highlighted in the 2024 KDIGO guidelines (Kidney Disease: Improving Global Outcomes (KDIGO) CKD Work Group, 2024; Kalantar-Zadeh et al., 2021). Current CKD management strategies broadly fall into two categories. The first involves conservative management for patients not yet on dialysis, centered on guideline-directed medical therapy (including RASI, SGLT2 inhibitors, MRA, and GLP-1 RAS) coupled with essential lifestyle modifications. Notably, the primary goal of conservative management is to slow disease progression and delay the initiation of renal replacement therapy; however, it cannot reverse established renal fibrosis. If the disease has progressed to ESRD, the second approach, renal replacement therapy *via* dialysis or transplantation, must be considered (Kalantar-Zadeh et al., 2021). Therefore, exploring novel therapeutic targets for renal fibrosis and developing innovative treatments holds significant clinical relevance.

Nephron injury may initiate a renal fibrotic process, including epithelial-mesenchymal transition (EMT) of tubular epithelial cells, activation and proliferation of myofibroblasts, and excessive ECM accumulation. These processes collectively contribute to irreversible tissue injury, manifested as glomerulosclerosis, tubulointerstitial fibrosis, and intrarenal vascular sclerosis. The histopathological features generally include loss and injury of intrinsic renal cells, infiltration of inflammatory cells, tubular atrophy and collapse, reduced vascular density, and progressive ECM accumulation. Ultimately, these alterations disrupt normal renal architecture and lead to irreversible loss of renal function (Ruiz-Ortega, Lamas & Ortiz, 2022). It is important to note that the conceptual framework of EMT has evolved considerably in recent years. The traditional view of EMT as a binary transition, in which epithelial cells fully convert into migratory mesenchymal cells, has been refined by recognizing that cells often adopt a partial epithelial-mesenchymal transition (pEMT) state during fibrogenesis (Sheng & Zhuang, 2020; Song et al., 2024). In this intermediate state, cells simultaneously express epithelial and mesenchymal markers and exhibit pro-fibrotic characteristics, such as increased extracellular matrix (ECM) production, without fully losing epithelial integrity (Naillat et al., 2024; Sheng & Zhuang, 2020). This refined understanding is particularly relevant to renal fibrosis, as tubular epithelial cells in fibrotic kidneys typically display partial EMT features rather than complete mesenchymal transformation (Sheng & Zhuang, 2020).

Mitogen-activated protein kinase 1 (MAPK1), a core member of the mammalian MAPK protein family, serves as a principal kinase within the extracellular signal-regulated kinase (ERK) signaling pathway. The MAPK family encompasses ERK (MAPK1-3), p38, and c-Jun N-terminal kinase (JNK) pathways. Despite differences in receptor activation and functional outcomes, these signaling pathways can act synergistically in disease progression (Kim & Choi, 2010). In the kidney, MAPK1/ERK2 is expressed in most primary renal cells, including tubular epithelial cells and podocytes, functioning as a critical signaling hub under both physiological (homeostasis) and pathological conditions. Studies indicate that MAPK1/ERK2 activation closely correlates with renal fibrosis progression, demonstrating sustained activity throughout the fibrotic process (Cheng et al., 2013; Nishida et al., 2008; Stambe et al., 2004). Upon upstream stimulation, MAPK1/ERK2 undergoes phosphorylation and activation, facilitating its nuclear translocation, where it orchestrates key transcription factors (Tao et al., 2023). Excessive activation of the MAPK1/ERK2 pathway promotes fibroblast activation, EMT, and excessive ECM deposition, eventually leading to irreversible renal dysfunction. Specific studies have clearly established a pro-fibrotic role for the MAPK1/ERK2 pathway in the kidney (Chen et al., 2016). Therapeutic targeting and inhibition of this pathway might slow renal fibrosis progression; however, the central role and therapeutic potential of the MAPK1/ERK2 pathway remain inadequately characterized. Accordingly, this review aims to clarify the key mechanisms by which MAPK1/ERK2 contributes to renal fibrosis and summarize recent findings regarding chemically synthesized drugs and natural bioactive compounds targeting this pathway. A noteworthy aspect is that, despite the high sequence homology between ERK1 and ERK2, and their frequent synergistic activation downstream of MEK1/2, the two isoforms differ substantially in their expression levels across cell types. This variation may result in the functional dominance of one isoform over the other under specific pathological conditions (Buscà, Pouysségur & Lenormand, 2016; Olea-Flores et al., 2019; Park, 2023). This review focuses on the MAPK1/ERK2 pathway, whose pivotal role in renal fibrosis has been well established. Building upon existing literature, we emphasize elucidating the functional differences between ERK1 and ERK2, which have important implications for the development of more precise isoform-selective therapeutic strategies. We anticipate that this multidimensional perspective will facilitate the development of future anti-fibrotic therapies. In this review, the term “synthetic drugs” refers to well-defined, chemically synthesized molecules (*e.g.*, DPP-4 inhibitors), whereas “natural bioactive compounds” denotes purified monomeric molecules derived from traditional medicinal herbs.

The main audience of this review includes researchers in the fields of kidney disease, pharmacology, and drug discovery. Following a comprehensive overview of the MAPK1/ERK2 signaling pathway and its targeted compounds, we aim to provide tailored insights for two distinct groups. For basic researchers, we present an overview of molecular mechanisms and recent advances in this field. For scholars engaged in translational research and drug development, including pharmaceutical chemists and pharmacologists, we provide evidence supporting target validation, detailed information on lead compounds (both synthetic and natural), and strategic perspectives to guide future research directions.

## Survey Methodology

To ensure a comprehensive overview of the literature, we conducted systematic searches in PubMed, Web of Science, and the China National Knowledge Infrastructure (CNKI) from database inception to March 20, 2026. The search strategy combined Boolean operators (AND/OR/NOT) with relevant keyword synonyms, covering renal fibrosis, the MAPK1/ERK2 signaling pathway, and therapeutic interventions. The search was conducted in two phases to balance broad coverage with targeted identification of therapeutic studies.

Phase 1: Broad search for MAPK1/ERK2 in renal fibrosis

The initial search aimed to identify all studies investigating the role of MAPK1/ERK2 signaling in renal fibrosis, without restricting intervention types. The representative PubMed search string was: (“erk1/2” OR “extracellular signal-regulated kinase 1/2” OR “MAPK3/MAPK1” OR “mitogen-activated protein kinase 3/1” OR “MAPK1” OR “ERK2”) AND (“renal fibrosis” OR “kidney scarring” OR “nephrogenic fibrosis” OR “renal interstitial fibrosis”).

Phase 2: Targeted search for therapeutic interventions

To complement the broad search and identify compounds targeting the MAPK1/ERK2 pathway in renal fibrosis, a secondary search was performed using terms related to therapeutic interventions. This approach captured studies investigating synthetic drugs, natural bioactive compounds, and other pharmacological agents. The search string was: (“renal fibrosis” OR “kidney fibrosis” OR “diabetic nephropathy” OR “diabetic kidney disease” OR “CKD” OR “tubulointerstitial fibrosis”) AND (“MAPK1” OR “ERK2” OR “extracellular signal-regulated kinase 2” OR “mitogen-activated protein kinase 1” OR “MAPK/ERK pathway” OR “ERK1/2” OR “p-ERK”) AND (“inhibitors” OR “antagonists” OR “drugs” OR “pharmacological” OR “natural products” OR “traditional Chinese medicine” OR “bioactive compounds” OR “flavonoid” OR “terpenoid” OR “saponin”).

Results from both phases were merged, and duplicates were removed. Reference lists of included studies and relevant reviews were manually screened to identify additional eligible studies not captured by electronic searches. Complete search strategies for Web of Science and CNKI are provided in Table S1.

Inclusion and exclusion criteria: To guide study selection, predefined inclusion and exclusion criteria were established (Table 1). These criteria ensured that selected studies directly or indirectly addressed the role of MAPK1/ERK2 in renal fibrosis or provided relevant mechanistic or pharmacological insights. The criteria were applied during screening to maintain focus and consistency, in line with the narrative nature of this review.

**Table 1:** Inclusion and exclusion criteria for literature.

Standard	Incorporate	Exclude
Research type	Original research articles (*in vivo*/*in vitro* experiments), review articles, systematic reviews	Case Reports, Meeting Abstracts, Commentaries, Editorials
Research subjects	The study directly addresses renal fibrosis and the MAPK1/ERK2 pathway	Studies did not investigate non-renal fibrosis or did not explore the MAPK1/ERK2 pathway
Intervention/Exposure	The study explicitly examines the effects of drugs (Synthetic Drugs/Natural Bioactive Compounds) on pathways	No drug intervention was involved (Synthetic Drugs/Natural Bioactive Compounds), or although drugs were involved, no indicators related to the MAPK1/ERK2 pathway were detected
Outcome indicator	Includes expression of pathway-related molecular markers (*e.g.*, p-ERK1/2, p-MEK) and renal fibrosis markers (*e.g.*, α-SMA, collagen)	No objective molecular, cellular, or pathological outcome markers associated with renal fibrosis or the MAPK1/ERK2 pathway were reported
Language	Chinese and English	Other non-Chinese and non-English literature
Time Range	From the establishment of the database to March 20, 2026	None

Literature screening: Two researchers (M.C. and Y.P.) independently screened titles and abstracts of retrieved records. Full texts of potentially eligible articles were subsequently assessed according to the criteria in Table 1. Disagreements were resolved through discussion or consultation with a third researcher (J.F.). A total of 143 articles were included in this narrative review, encompassing both mechanistic and pharmacological studies. Priority was given to studies investigating single compounds with clearly defined chemical structures, consistent with the journal’s emphasis on mechanistic clarit. Although traditional compound formulations remain an important source for drug discovery, such studies were excluded due to two main limitations: difficulty in identifying active components and the lack of direct comparisons with purified compounds. These limitations hinder precise mechanistic interpretation and were therefore considered incompatible with the scope of this review.

## Regulatory Mechanisms of the MAPK1/ERK2 Signaling Pathway in Renal Fibrosis

### Activation mechanism of the MAPK1/ERK2 signaling pathway

**Figure 1:**
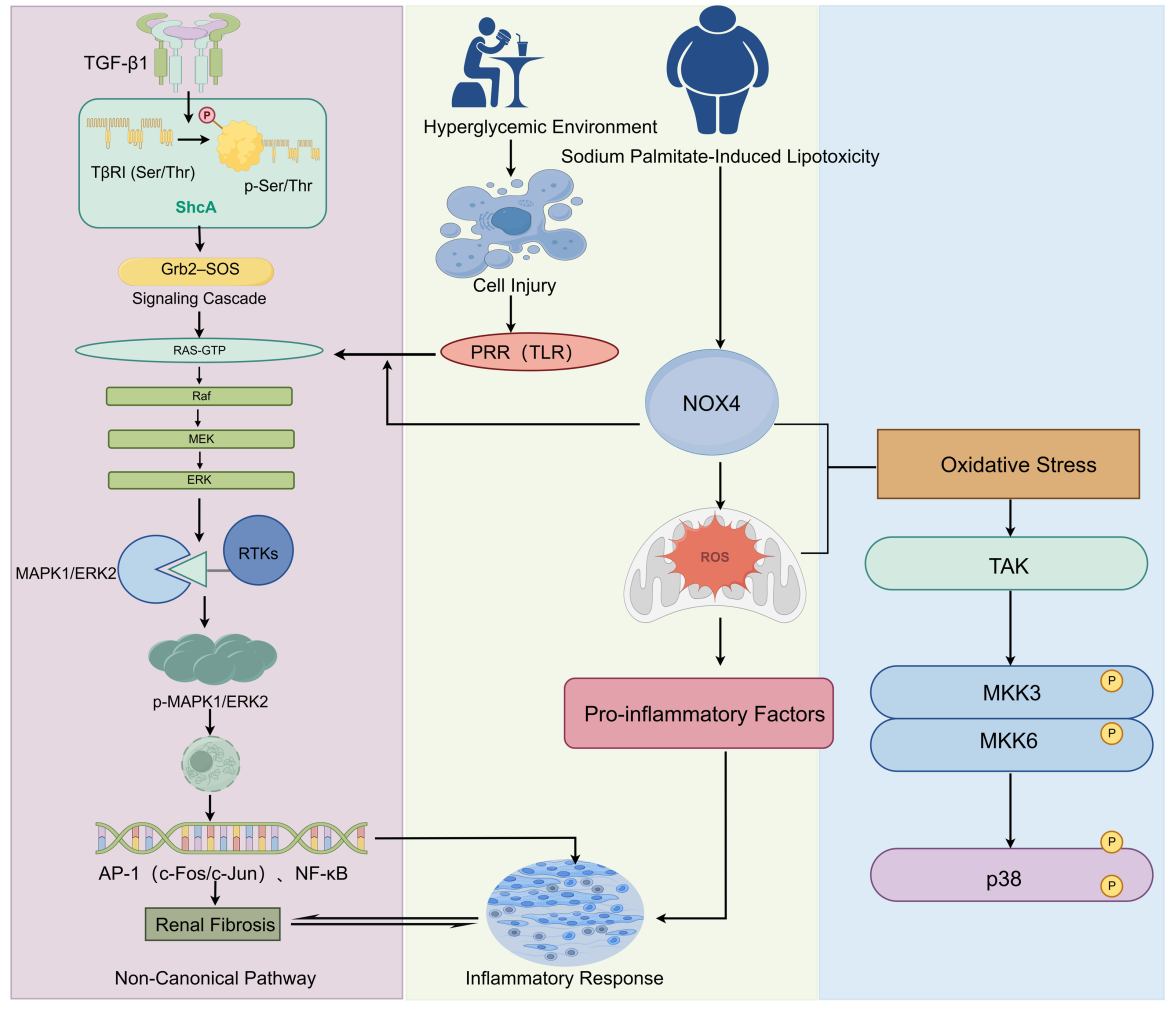
Activation mechanisms of the MAPK1/ERK2 signaling pathway in renal fibrosis. The major upstream stimuli and signaling cascades leading to MAPK1/ERK2 activation and subsequent fibrotic responses. Three primary activating pathways are shown: (1) TGF-β1 pathway: TGF-β1 activates the non-canonical pathway *via* TβRI-mediated phosphorylation of ShcA, which recruits the Grb2–SOS complex to activate RAS–GTP, followed by sequential activation of Raf, MEK, and ERK. This pathway operates independently of Smad signaling. (2) Hyperglycemia and inflammatory pathway: High glucose and inflammatory factors act through pattern recognition receptors (PRRs), notably Toll-like receptors (TLRs), to trigger the Ras/Raf/MEK/ERK cascade, leading to phosphorylation and nuclear translocation of MAPK1/ERK2, which subsequently activates transcription factors such as AP-1 and NF-κ B. (3) Oxidative stress and lipotoxicity pathway: Sodium palmitate-induced lipotoxicity upregulates NOX4, promoting reactive oxygen species (ROS) accumulation. ROS oxidatively inactivate phosphatases while activating upstream kinases (*e.g.*, Raf, MEK), ultimately driving MAPK1/ERK2 phosphorylation. Activated MAPK1/ERK2 phosphorylates downstream effectors including p38 MAPK, MKK3/6, and transcription factors (AP-1, NF-κ B), thereby promoting inflammatory responses, cell injury, and renal fibrosis. The “non-canonical pathway” refers to TGF-β1 signaling that is independent of Smad but involves direct ShcA phosphorylation. Abbreviations: AP-1, activator protein-1; Grb2, growth factor receptor-bound protein 2; MAPK, mitogen-activated protein kinase; MEK, mitogen-activated protein kinase kinase; MKK3/6, mitogen-activated protein kinase kinase 3/6; NF-κ B, nuclear factor kappa-light-chain-enhancer of activated B cells; NOX4, NADPH oxidase 4; p38, p38 mitogen-activated protein kinase; PRR, pattern recognition receptor; Raf, rapidly accelerated fibrosarcoma; RAS, rat sarcoma virus; ROS, reactive oxygen species; RTKs, receptor tyrosine kinases; ShcA, Src homology 2 domain-containing transforming protein A; SOS, son of sevenless; TAK, transforming growth factor-β-activated kinase; TβRI, transforming growth factor-β type I receptor; TGF-β1, transforming growth factor-β1; TLR, Toll-like receptor. Created using Figdraw (http://www.figdraw.com).

MAPK1 (ERK2), a core member of the ERK signaling pathway within the MAPK family, is primarily activated during renal fibrosis by transforming growth factor-β1 (TGF-β1), hyperglycemia, and inflammatory stimuli. Once activated, MAPK1 triggers downstream signaling cascades that drive fibroblast activation, renal tubular epithelial-mesenchymal transition (EMT), mesangial matrix expansion, and excessive ECM deposition (Kim et al., 2007; Liu et al., 2024; Martin-Vega & Cobb, 2023) (Fig. 1).

#### Mechanism of TGF-β1-induced MAPK1/ERK pathway activation

TGF-β1, a critical mediator of renal fibrosis, exerts its effects on renal tubular epithelial cells by stimulating pro-fibrotic factor secretion. Research indicates that TGF-β1 activates MAPK1/ERK2 independently of Smad signaling through recruitment of tyrosine kinase pathways (Lee et al., 2007). Although the TGF-β type I receptor (TβRI) primarily functions as a serine/threonine kinase, studies have revealed that TβRI can directly phosphorylate the adaptor protein ShcA at serine/threonine residues. This phosphorylation activates the non-canonical Ras-Raf-MEK-ERK pathway *via* the ShcA-Grb2-SOS signaling axis, thereby coupling receptor tyrosine kinases (RTKs) to MAPK1/ERK2 kinases (Lee et al., 2007). Consequently, MAPK1/ERK2 undergoes phosphorylation and activation. Importantly, this process does not involve Smad signaling, highlighting a distinct, non-canonical mechanism through which TGF-β receptors regulate the MAPK pathway.

#### Mechanism of MAPK1/ERK pathway activation induced by high glucose and inflammatory factors

Exposure to high glucose directly induces phosphorylation and activation of MAPK1/ERK2 in renal tubular cells (Liu et al., 2024). Consistent with this finding, studies using diabetic mouse models demonstrate increased MAPK activity, particularly elevated ERK and JNK phosphorylation, in renal tissues (Jiao et al., 2015). Hyperglycemia-induced chronic inflammation appears to be a primary driver of MAPK signaling pathway activation, including MAPK1/ERK2 (Han et al., 2022). Injury from high glucose activates an inflammatory cascade driven by pattern-recognition receptors (PRRs), particularly Toll-like receptors (TLRs) and specific cytokine receptors. These receptors trigger the downstream Ras-Raf-MEK-ERK signaling cascade, enhancing inflammatory responses (Roux & Blenis, 2004). Activation of MAPK1/ERK2 results in nuclear translocation and stimulation of transcription factors such as AP-1 (c-Fos/c-Jun) and NF-κB (Chen et al., 2018; Lund et al., 2025).

#### Mechanisms of MAPK1/ERK pathway activation induced by oxidative stress and lipotoxicity

Activation of the MAPK1/ERK2 pathway is mechanistically related to NADPH oxidase 4 (NOX4), a key regulator of oxidative stress. Up-regulation of NOX4 leads to reactive oxygen species (ROS) accumulation, triggers pro-inflammatory factor release, and amplifies inflammatory responses (Yoo et al., 2020). Sodium palmitate induces lipotoxicity, disrupts cellular lipid metabolism, and aggravates fibrosis in human mesangial cells (Su et al., 2019). Studies of lipotoxicity indicate that metabolic stressors like sodium palmitate promote ROS generation through NOX4. ROS accumulation oxidatively inactivates specific phosphatases while activating upstream kinases, such as Raf and MEK, ultimately resulting in MAPK1/ERK2 phosphorylation (p-ERK) (Chen et al., 2020; Zhang et al., 2025c). Activation of downstream pathways then upregulates fibrosis-associated genes (*e.g.*, α-SMA and collagen), exacerbating renal fibrosis and inflammation (Cheng et al., 2016). Therefore, in human mesangial cells (HMCs), the NOX4/MAPK1/ERK2 pathway constitutes a central mechanism of lipotoxicity-induced fibrosis (Chen et al., 2020). In experimental glomerulonephritis, oxidative stress, closely associated with NOX4 upregulation, is predominantly mediated through activation of the p38 MAPK pathway, promoting pathogenic mesangial cell proliferation (Gao et al., 2024; Lee et al., 2012). This suggests distinct stimuli preferentially activate specific MAPK pathways (*e.g.*, oxidative stress favors p38 MAPK, while lipotoxicity favors ERK), or that multiple pathways may act synergistically through NOX4.

### Fibrogenic mechanism of the MAPK1/ERK2 signaling pathway

**Figure 2:**
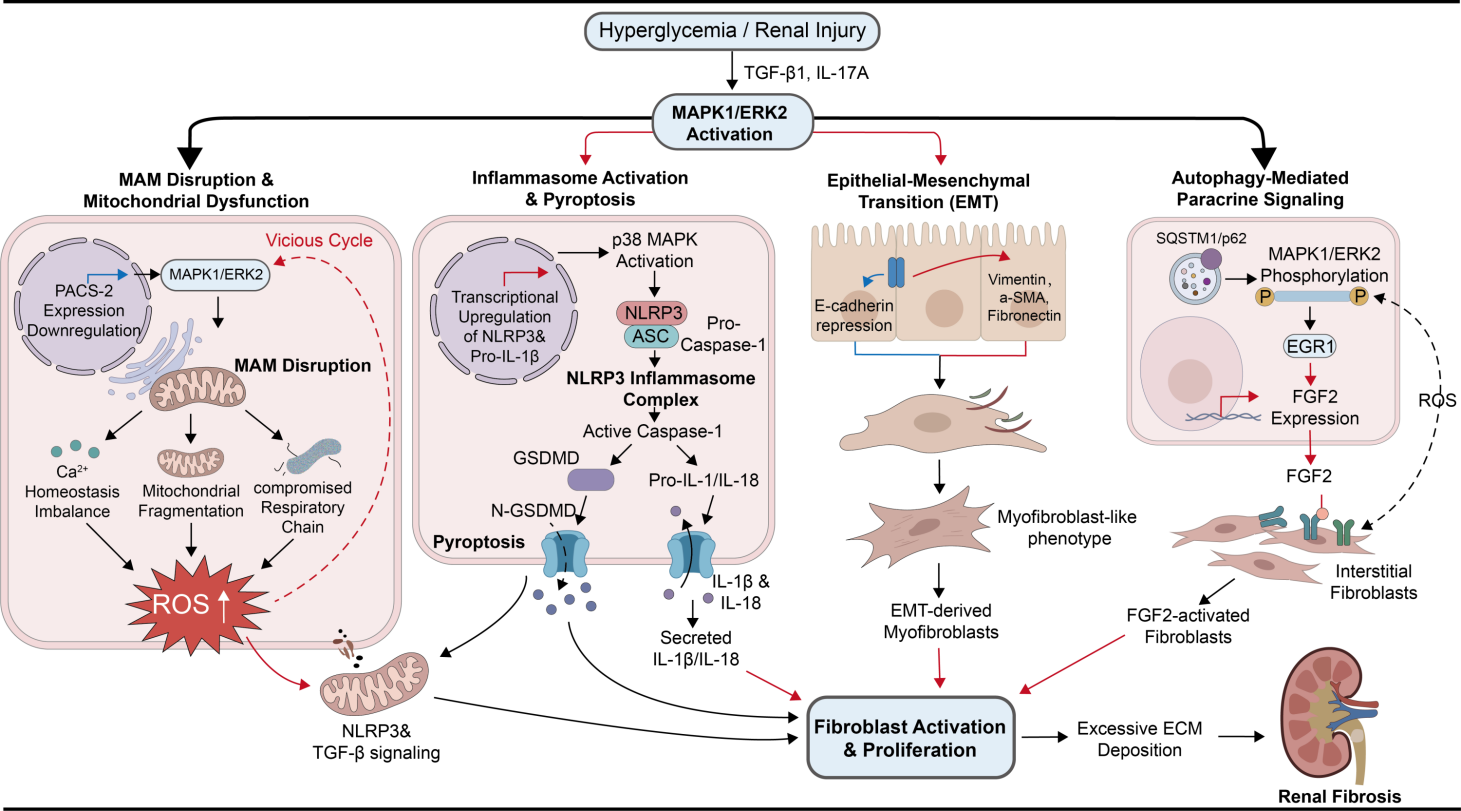
Fibrogenic mechanisms of the MAPK1/ERK2 signaling pathway in renal fibrosis. Four major pro-fibrotic mechanisms mediated by MAPK1/ERK2 activation: (1) MAM disruption and mitochondrial dysfunction; (2) inflammasome activation and pyroptosis; (3) epithelial-mesenchymal transition (EMT); and (4) autophagy-mediated paracrine signaling. These interconnected pathways converge on fibroblast activation, excessive ECM deposition, and ultimately renal fibrosis. Abbreviations: MAPK1/ERK2, Mitogen-Activated Protein Kinase 1/Extracellular Signal-Regulated Kinase 2; TGF-β1, Transforming Growth Factor Beta 1; IL-17A, Interleukin 17A; IL-1β, Interleukin 1 Beta; IL-18, Interleukin 18; MAM, Mitochondria-Associated Endoplasmic Reticulum Membrane; PACS-2, Phosphofurin Acidic Cluster Sorting Protein 2; ROS, Reactive Oxygen Species; NLRP3, NOD-Like Receptor Family Pyrin Domain Containing 3; ASC, Apoptosis-Associated Speck-Like Protein Containing a CARD; GSDMD, Gasdermin D; N-GSDMD, N-Terminal Fragment of Gasdermin D; EMT, Epithelial-Mesenchymal Transition; α-SMA, Alpha-Smooth Muscle Actin; SQSTM1/p62, Sequestosome 1/p62; EGR1, Early Growth Response 1; FGF2, Fibroblast Growth Factor 2; ECM, Extracellular Matrix.

Abnormal activation of the MAPK1/ERK2 signaling pathway is a critical factor driving renal fibrosis. Recent studies (Guo et al., 2025b; Juan et al., 2023; Liu et al., 2024) have identified four primary mechanisms through which MAPK1/ERK2 signaling promotes fibrosis: (1) Mediating mitochondria-associated membrane (MAM) disruption and subsequent mitochondrial dysfunction; (2) activating inflammasomes and increasing the release of inflammatory cytokines to enhance inflammation; (3) directly regulating the initiation and progression of EMT; and (4) functioning downstream of autophagy to induce paracrine fibroblast activation *via* the EGR1-FGF2 axis. Persistent activation of MAPK1/ERK2 leads to structural damage and loss of renal function through these interconnected pathways, making it a crucial therapeutic target in renal fibrosis (Fig. 2).

#### MAPK1/ERK2-mediated MAM disruption and mitochondrial dysfunction

In diabetic kidney disease (DKD), hyperglycemia elevates MAPK1/ERK2 expression in renal tubules, which subsequently downregulates phosphofurin acidic cluster sorting protein 2 (PACS-2), impairing MAM integrity and causing mitochondrial dysfunction (Liu et al., 2024). This mechanism may represent a central element of the MAPK1/ERK2 pathway’s profibrotic effects. The MAM is a dynamic interface consisting of multiple proteins, involved in mitochondrial homeostasis, lipid metabolism, and calcium ion regulation (Perrone et al., 2020). PACS-2, the first identified MAM-resident protein and a critical regulator of MAM structure, is highly expressed in renal tubules (Liu et al., 2024; Zhao et al., 2017). PACS-2 overexpression protects against high glucose-induced MAM disruption, thereby mitigating DKD progression. Studies indicate that elevated MAPK1/ERK2 levels induced by hyperglycemia reduce PACS-2 expression, disrupt the MAM structure, and induce mitochondrial fragmentation (Liu et al., 2024). These effects are reversible by treatment with the MAPK1/ERK2 inhibitor VX-11e or PACS-2 overexpression (Liu et al., 2024). Current evidence supports MAPK1′s negative regulatory effect on PACS-2 expression and complex assembly, although direct phosphorylation of PACS-2 by MAPK1 remains unconfirmed (Liu et al., 2024).

Impaired MAM integrity leads to severe mitochondrial dysfunction, disrupting calcium transport at the endoplasmic reticulum-mitochondria interface and disturbing cellular calcium homeostasis. This disruption damages the mitochondrial respiratory chain and causes imbalance in mitochondrial dynamics, shifting towards fragmentation. Additionally, MAM damage promotes excessive ROS generation, exacerbating oxidative stress and cellular injury (Fernandes et al., 2021; Mao et al., 2022; Wang et al., 2022b). Such mitochondrial dysfunction accelerates renal fibrosis by facilitating renal tubular epithelial cell transformation into interstitial fibroblasts *via* activation of downstream pathways such as the NOD-like receptor thermal protein domain-associated protein 3 (NLRP3) inflammasome and TGF-β signaling. These pathways drive excessive ECM deposition, further aggravating fibrosis (Sun et al., 2024). Importantly, a vicious cycle exists between MAPK1/ERK2 activation and mitochondrial dysfunction: mitochondrial ROS activate MAPK1/ERK2 signaling, which in turn amplifies mitochondrial damage, creating a positive feedback loop perpetuating cellular injury (Liu et al., 2024; Yang et al., 2023).

#### MAPK1/ERK2-mediated activation of inflammasomes and inflammatory cytokine release

The MAPK1/ERK2 pathway significantly amplifies inflammation by indirectly facilitating activation of the NLRP3 inflammasome, either through induction of oxidative stress or cooperative signaling with p38 MAPK. Activation of the inflammasome results in the secretion of pro-inflammatory and pro-fibrotic cytokines such as IL-1β and IL-18, sustaining a pathological cycle of renal inflammation and fibrosis acceleration (Guo et al., 2025b). Extensive evidence demonstrates that p38 MAPK closely interacts with NLRP3 inflammasome activation and Gasdermin D (GSDMD)-mediated pyroptosis. Inhibition of p38 reduces expression of NLRP3 and GSDMD-N, alleviating acute inflammatory injury (Wang et al., 2024). Studies using (Zhen et al., 2023) a unilateral ureteral obstruction (UUO) mouse model showed significant correlations between renal MAPK1/ERK2 activation and NLRP3 inflammasome expression (Vervaeke & Lamkanfi, 2025). Inhibition of MAPK1/ERK2 suppressed inflammasome activation and mitigated fibrosis. Additionally, the NLRP3 inflammasome is a downstream target of ERK signaling; inhibiting ERK decreases expression of NLRP3 and caspase-1 precursor proteins (Wang et al., 2022a). Notably, existing studies clearly indicate that regulation of NLRP3 inflammasomes by p38 MAPK or MAPK1/ERK2 is indirect (Guo et al., 2025b; Jenster et al., 2023; Wang et al., 2024). In contrast, Bruton’s tyrosine kinase (BTK) has been demonstrated to directly phosphorylate NLRP3 (Weber, 2021). Although direct interactions cannot be completely excluded under certain physiological or pathological contexts, no conclusive experimental evidence currently supports direct NLRP3 phosphorylation by p38 MAPK or ERK2. Activated NLRP3 inflammasomes recruit apoptosis-associated speck-like protein containing a CARD (ASC) and caspase-1 to form inflammasome complexes. Subsequently, activated caspase-1 cleaves GSDMD, releasing its N-terminal domain (GSDMD-N), forming membrane pores, and facilitating release of inflammatory cytokines IL-1β and IL-18 (Elias, Lyons & Muruve, 2023). Activated caspase-1 also cleaves the precursors of IL-1β and IL-18 into mature, biologically active cytokines. These cytokines directly promote fibroblast proliferation and ECM accumulation, thereby exacerbating renal fibrosis (Kahlenberg & Kang, 2020).

The synergistic yet distinct roles of MAPK1/ERK2 and p38 MAPK in inflammation have been characterized. Current evidence indicates that MAPK1/ERK2 primarily mediates transcriptional upregulation of NLRP3 and pre-IL-1β (Gong et al., 2022), while p38 MAPK may play a complementary role, and p38 MAPK is directly involved in inflammasome assembly and caspase-1 activation (Wang et al., 2024). This functional specialization provides a mechanistic rationale for the enhanced efficacy of dual inhibition. Accordingly, therapeutic strategies targeting both MAPK1/ERK2 and p38 MAPK may achieve more effective suppression of renal inflammation and fibrosis.

#### Regulation of EMT by MAPK1/ERK2

Previous studies have demonstrated that MAPK1/ERK2 plays a critical role in renal fibrosis by inducing EMT. Following kidney injury, pro-fibrotic factors such as TGF-β1 and IL-17A activate MAPK1/ERK2, leading to suppression of E-cadherin expression in tubular epithelial cells and upregulation of mesenchymal markers, including vimentin, α-SMA, and fibronectin (Juan et al., 2023). Disruption of epithelial polarity and intercellular junctions represents an early event that facilitates cell migration. Subsequently, cells undergo phenotypic transition and acquire myofibroblast-like properties, contributing to pathological ECM deposition and progressive renal interstitial fibrosis (Rhyu et al., 2005).

Targeted inhibition of ERK1/2 has been shown to reverse EMT in both *in vivo* and *in vitro* models. In a UUO mouse model, inhibition of the ERK1/2 pathway using PD98059 (a MEK1/2 inhibitor) or specific siRNA restored E-cadherin expression, and significantly reduced α-SMA expression and collagen synthesis (Wu et al., 2016). These effects alleviated tubular structural damage and interstitial fibrosis, demonstrating that central role of ERK1/2 in EMT regulation.

#### Autophagy-mediated activation of MAPK1/ERK2 and its profibrotic consequences

Recent evidence has identified a novel pro-fibrotic mechanism involving the interaction between autophagy and the MAPK1/ERK2 signaling pathway. Livingston et al. (2024) demonstrated that in the maladaptive repair phase following renal ischemia-reperfusion injury, autophagy promotes fibroblast activation through MAPK/ERK-dependent induction of fibroblast growth factor-2(FGF2). This finding links intracellular degradation processes with paracrine signaling between injured tubular cells and interstitial fibroblasts.

Mechanistically, the autophagy adaptor protein SQSTM1/p62 interacts with MAPK/ERK and facilitates its phosphorylation (Livingston et al., 2024). Activated ERK subsequently upregulates the transcription factor early growth response 1 (EGR1), which binds directly to the FGF2 promoter and drives its expression. FGF2 is then secreted by tubular epithelial cells and acts in a paracrine manner to stimulate fibroblast activation and ECM deposition. This signaling cascade establishes a direct molecular link between tubular cell autophagy and fibroblast-mediated fibrogenesis (Livingston et al., 2024).

Importantly, this profibrotic cascade is abrogated under autophagy-deficient conditions. Tubule-specific autophagy related 7(Atg7) knockout mice exhibit accumulation of SQSTM1/p62, which sequesters MAPK/ERK and prevents its activation. This sequestration reduces EGR1 and FGF2 expression and consequently attenuates renal fibrosis. These findings highlight the dual role of autophagy in kidney repair: while basal autophagy maintains cellular homeostasis, sustained autophagy during the repair phase may exacerbate fibrosis through the MAPK1/ERK2-EGR1-FGF2 signaling axis (Yang et al., 2025).

This autophagy-mediated mechanism may also intersect with other profibrotic pathways described above. For instance, mitochondrial ROS generated by MAPK1/ERK2-induced MAM disruption may further enhance autophagy, creating a positive feedback loop that amplifies fibrotic signaling (Liu et al., 2024). This potential crosstalk suggests that the autophagy-MAPK1/ERK2 axis operates not only independently but also in coordination with other pro-fibrotic networks.

Consistent with this concept, multiple studies have demonstrated that targeting the interaction between autophagy and MAPK signaling exerts antifibrotic effects (Tu et al., 2026). For instance, rhein has been shown to inhibit autophagy by modulating the adenosine 5′-monophosphate (AMP)-activated protein kinase (AMPK/mTOR) and ERK/p38 MAPK signaling pathways, thereby alleviating renal tubular injury and fibrosis (Tu et al., 2017). Collectively, these findings position the autophagy-MAPK1/ERK2 axis as both a key driver of renal fibrosis and a promising therapeutic target.

## Bioactive Compounds Targeting the MAPK1/ERK2 Pathway

Building on the mechanistic framework of the MAPK1/ERK2 pathway, this section examines compounds with therapeutic potential for its modulation. To ensure mechanistic clarity, the discussion is organized into two categories: synthetic drugs and natural bioactive compounds. Both groups comprise well-defined chemical entities, enabling precise attribution of their antifibrotic effects, in contrast to the complexity of multi-component herbal formulations. The chemical structures and CAS numbers of all compounds identified through the systematic search (summarized in Tables 2 and 3) are presented in Table S2.

**Table 2:** Synthetic drugs targeting the MAPK1/ERK2 pathway.

Medication name	Specific dosage/route of administration	Mechanism of action	Effects on the ERK2 pathway	Effects on renal fibrosis	Experimental model	Development status	Potential toxicity	References
Linagliptin	5 mg/kg/day, orally	Inhibits Smad, ERK, and p38 MAPK phosphorylation, downregulates HIF-1α and LOXL2 signaling	Direct and indirect inhibition	Inhibit EMT, reduce protein expression of epithelial, mesenchymal, and transcriptional biomarkers, and delay renal fibrosis.	Chronic kidney injury in rats induced by tacrolimus (TAC)	Approved for marketing: For patients with type 2 diabetes	nasopharyngitis, diarrhea, cough, headache, *etc.*	Nady, Abd El-Raouf & El-Sayed (2024)
Vildagliptin	10.6 ± 1.5 mg/kg/day, Administer with drinking water.	Inhibits ERK and 4-HNE expression, reduces IL-6 and COX-1 mRNA accumulation, and enhances HO-1 mRNA expression.	Indirect inhibition	Reduce oxidative stress and alleviate renal fibrosis.	UUO mice	Approved for marketing: For patients with type 2 diabetes	Headache, nasopharyngitis, cough, constipation, dizziness, excessive sweating	Honma et al. (2024)
Pirfenidone	*In vivo*: 500 mg/kg/day, 0.5% carboxymethylcellulose solution suspension administered *via* gavage *in vitro*: TGF-β1 (5 ng/ml) + pirfenidone (0.2 or 0.5 mg/ml) cultured for 24 h	Inhibits P-ERK expression without affecting total ERK levels	Direct inhibition	Inhibits EMT and alleviates renal fibrosis. *in vitro*: Reduces expression of α-SMA, type I and III collagen, S100A4, and fibronectin, while increasing E-cadherin expression. *in vivo*: Reduces expression of transforming growth factor-β1, type III collagen, α-SMA, S100A4, and fibronectin, while increasing E-cadherin expression.	Human proximal tubule epithelial cells (HK-2), Ureteropelvic junction obstruction (UPJO) rats	Approved for marketing: For the treatment of idiopathic pulmonary fibrosis	Elevated urinary creatinine levels in rats, gastrointestinal reactions, skin disorders, hepatotoxicity, neurological disorders	Li et al. (2017)
Trametinib	*In vivo*: 3 mg/kg, administered orally once daily (100 mL per animal) *in vitro* experiment: Incubate with trametinib (10 or 20 nM) for 30 min.	Inhibits ERK1/2 and AKT phosphorylation, blocks UUO-induced mTORC1 activation and NOX4 overexpression, and suppresses STAT3, NF-κB, and Smad2/3 phosphorylation along with macrophage infiltration.	Direct inhibition	Improves collagen deposition and myofibroblast expansion during renal fibrosis. Reduces expression of vimentin and ASMA.	Mice treated with UUO or fed an adenine-enriched diet—as well as in cultured human primary fibroblasts.	Currently marketed: Currently used for the treatment of melanoma	Fever, chills, fatigue, peripheral edema, skin and subcutaneous tissue disorders, gastrointestinal disorders, *etc.*	Andrikopoulos et al. (2019)
Erlotinib	Erlotinib (EGFR tyrosine kinase inhibitor) therapy, 20 mg/kg/day, Oral, administered *via* gastric capsule.	Inhibition of EGFR phosphorylation (downstream ERK axis inhibition)	Indirect inhibition	Reduce expression of alpha-smooth muscle actin (α-SMA) and mRNA expression of fibrotic components (collagen I and III) and pro-fibrotic factors (TGF-β, connective tissue growth factor), thereby alleviating tubulointerstitial fibrosis.	eNOS−/− db/db mice, adenine/other chronic diabetic nephropathy mouse models	Approved: For use as third-line therapy in patients with locally advanced or metastatic non-small cell lung cancer who have failed two or more prior chemotherapy regimens.	Rash, diarrhea, abnormal liver function, eye disorders (keratitis, conjunctivitis), *etc.*	Li et al. (2018)
Aronitin	*In vivo*: 1 mg/kg/day, intraperitoneal injection; *in vitro*: (2 μM) 4-hour incubation + TGFβ1 incubation for 48 h	Inhibits the TGFβ-1-mediated ERK-AKT signaling pathway and suppresses ERK2 phosphorylation.	Direct inhibition	Reduce renal tubular injury, extracellular matrix deposition in renal tubules, and the expression of alpha-smooth muscle actin, transforming growth factor-beta 1, and cytoplasmic inflammatory factors both *in vivo* and *in vitro*.	HK-2 cells, UUO mice, folic acid-induced renal injury mice (FA mice)	Approved (anti-cancer): Anti-renal fibrosis is in the preclinical exploration phase.	Fatigue, hypertension, skin toxicity reactions	Han et al. (2018) and Sun et al. (2023)
CaMKII Inhibitory Peptide AIP	*In vivo*: 0.625 mg/kg/d or 2.5 mg/kg/d, subcutaneous injection; *in vitro*: 25 μM, 50 μM, 100 μM for 24 h	Inhibits CaMKII phosphorylation, thereby suppressing EMT-associated TGF-β1/Smad2 and RAF/ERK signaling pathways.	Indirect inhibition	Inhibits the expression of fibronectin, type I collagen, matrix metalloproteinase-2, and alpha-smooth muscle actin both *in vivo* and *in vitro*; reduces TGF-β expression *in vivo*.	Mouse embryonic fibroblasts (NIH-3T3), HK-2 cells, and rat proximal tubule epithelial cells (NRK52E), UUO mice	Not yet listed: Anti-fibrosis is in the preclinical exploration phase.		Feng et al. (2023)
Fluphenidone	*In vivo*: 100 mg/(kg d), oral administration; *in vitro*: 2 mM/24-hour culture	By inhibiting ERK phosphorylation, reduce NADPH oxidase-mediated ROS production.	Direct inhibition	*In vitro*: Reduces Angi-induced expression of ROS, NOX2, fibronectin, type I collagen (A1), and p-ERK. *in vivo*: Decreases expression of type I collagen (A1) and fibronectin, while downregulating NOX2, MDA, and p-ERK expression. Inhibits oxidative stress and alleviates renal fibrosis.	UUO rats, NRK52E cells	Not yet listed (clinical research stage): Anti-liver fibrosis has entered Phase III clinical trials.	Hepatotoxicity, photosensitivity	Qin et al. (2015)
Urolithin A	*In vivo*: 10, 20, 40 mg/kg/day orally/intravenously *in vitro*: 5, 10, 25, 50, 100, and 200 μM for 24 h	Partial inhibition of ERK1/2, JNK1/2, and p38 phosphorylation	Direct inhibition	*In vitro*: Inhibits fibrosis and proliferation in HK-2 cells; reduces expression of TGF-β1, p-Smad3, and p-P38/JNK/ERK; increases expression of Smad7. *in vivo*: Improves renal tissue injury, reduces macrophage infiltration and proinflammatory cytokine expression; decreases TGF-β1, p-Smad2/3, and p-P38/JNK/ERK expression while increasing Smad7 expression.	HK-2 cells, UUO rats	Not yet listed: Anti-fibrosis is in the preclinical exploration phase.		Cheng et al. (2021)
Risedronate (RIS)	*In vivo*: 5 μg/kg, subcutaneous injection (s.c.), twice weekly, for 21 consecutive days (administration began immediately after surgery); *In vitro*: 10 μM; NRK-52E cells were co-treated with rh-TGF-β1 (10 ng/mL) for 48 h.	Nitrogen-containing bisphosphonates inhibit protein prenylation by blocking farnesyl-tetraphosphate synthase (FPPS). This, in turn, inhibits the activation of the downstream RhoA/ROCK1 signaling pathway.	Indirect inhibition	In UUO rats, RIS treatment reduced collagen deposition, fibronectin levels, and hydroxyproline content in renal tissue; downregulated mRNA and protein expression of TGF-β, CTGF, collagen I, and fibronectin; and improved tubular damage and glomerulosclerosis.	*In vivo*: Unilateral ureteral obstruction (UUO) model in male SD rats. *in vitro*: Fibrosis model induced in normal rat kidney epithelial cells (NRK-52E) by treatment with rh-TGF-β1 (10 ng/mL) for 48 h.	Approved drug (for osteoporosis); currently in preclinical development for renal fibrosis (in the exploratory drug repurposing phase).	RIS is primarily excreted *via* the kidneys; therefore, dose optimization and monitoring of renal function are essential in patients with chronic kidney disease (CKD). When used clinically in patients with impaired renal function, caution is warranted due to the potential risk of nephrotoxicity.	Bhadange & Gaikwad (2025)
Metformin	*In vivo*: 200 mg/kg/day, administered by gavage for 7 consecutive days *in vitro*: 1 mM; after 1-hour pretreatment, NRK-49F cells were co-treated with Ang II (1 μM) for 24–48 h.	Metformin significantly reduced the p-ERK/total ERK ratio and inhibited the phosphorylation-mediated activation of the ERK signaling pathway; this effect was associated with downregulation of TGF-β and a reduction in ECM proteins.	Direct/indirect inhibition	Reduce collagen deposition; decrease the protein expression of fibronectin (FN), collagen I (Col-I), and TGF-β; and inhibit the excessive production of ECM proteins in Ang II-induced fibroblasts.	*In vivo*: Unilateral ureteral obstruction (UUO) model in male C57BL/6J mice. *in vitro*: Rat renal fibroblasts (NRK-49F) stimulated with Ang II (1 μM).	Approved drug (for type 2 diabetes); currently in preclinical development for renal fibrosis	No significant toxicity was reported at the doses used in this study.	Shen et al. (2016)
Erythropoietin (EPO)	*In vivo*: 100 U/kg, intraperitoneal injection (i.p.), once daily for 7 consecutive days. *in vitro*: 10U/mL, 24 h treatment; HK-2 cells were co-treated with rhTGF-β1 (10 ng/mL).	By inhibiting the expression of miR-21-5p, its suppression of the target gene SPRY1 is lifted, thereby blocking the activation of the downstream ERK/NF-κB signaling pathway.	Indirect inhibition	Reduced collagen deposition and collagen fraction; decreased mRNA and protein expression of α-SMA and collagen I; lowered BUN and SCr levels; reduced MPO-positive inflammatory cell infiltration and expression of IL-6 and TNF-α.	*In vivo*: Unilateral ureteral obstruction (UUO) model in male C57BL/6 mice. *in vitro*: Fibrosis model induced in human proximal renal tubular epithelial cells (HK-2) by treatment with rhTGF-β1 (10 ng/mL) for 24 h.	Approved drugs (for renal anemia and related anemias); in preclinical development for renal fibrosis	Potential side effects include high blood pressure and an increased risk of blood clots	Liu et al. (2026)
SHP099 (SHP2 inhibitor)	*In vivo*: 7.5 mg/kg, intraperitoneal (i.p.) injection, once daily, for 4 consecutive weeks. *In vitro*: 10 μM, pretreated for 3 h, followed by co-incubation of HK-2 cells with LPS (20 μg/mL) for 24 h.	Inhibits the phosphorylation-mediated activation of SHP2, thereby blocking the activation of downstream ERK/NF-κB signaling pathways	Indirect inhibition	Reduces the mRNA expression of TGF-β, FN, and COL-1 in renal tissue; improves pathological changes in renal tissue	*In vivo*: Female MRL/lpr lupus nephritis mice (spontaneous lupus model) *In vitro*: Human renal proximal tubule epithelial cells (HK-2)	Not yet on the market: Currently in preclinical research	No significant toxicity was reported; SHP099, as a selective inhibitor, was well tolerated at the doses used.	Chen et al. (2026)
Mefunidone (MFD)	*In vivo*: 25, 50, or 100 mg/kg/day, administered by gavage once daily for 3, 7, or 14 consecutive days. *In vitro*: 0.06–0.18 mM; pre-treated for 1–24 h, co-treated with TNF-α (10 ng/mL) in HK-2 cells, or co-treated with LPS (250 ng/mL) in primary mouse peritoneal macrophages.	By inhibiting T-cell and macrophage infiltration as well as the release of inflammatory cytokines (MCP-1, IL-6, TNF-α), it synergistically suppresses the phosphorylation and activation of three signaling pathways—ERK1/2, NF-κB (IκB phosphorylation), and STAT3 (Ser727)—thereby blocking the inflammation-fibrosis cascade through multiple targets.	Indirect inhibition	Alleviates tubulointerstitial injury; reduces mRNA and protein expression of collagen I and collagen III; decreases expression of α-SMA and fibronectin (FN); inhibits TGF-β1-induced proliferation and activation of renal fibroblasts (NRK-49F).	*In vivo*: Male SD rat UUO model *In vitro*: Human renal proximal tubule epithelial cells (HK-2); primary mouse peritoneal macrophages; rat renal fibroblasts (NRK-49F).	Preclinical studies (structurally optimized derivatives of fluphenidone; acute toxicity tests have been completed, showing lower toxicity than PFD and AKF-PD).	Acute and chronic toxicity studies have shown that mefunidone is less toxic than pirfenidone (PFD) and flufenidone (AKF-PD).	Liu et al. (2015)
Olmesartan	*In vivo*: 5 mg/kg/day, administered by gavage once daily for 28 consecutive days.	By blocking AT-1R, it inhibits the phosphorylation and activation of downstream MAPK cascades (ERK1/2, p38 MAPK, and JNK); simultaneously, it upregulates the expression of renal ACE-2 and Ang-(1-7)/Mas receptor proteins, thereby restoring RAS balance.	Indirect inhibition	Reduce the area of renal tissue fibrosis; decrease the expression of collagen III and fibronectin; downregulate TGF-β1 expression; alleviate tubular cell hypertrophy; and reduce the expression of the oxidative stress marker gp91-phox.	*In vivo*: Male C57BL/6J mice, an STZ-induced model of type 1 diabetic nephropathy.	Approved drug (for hypertension); currently in preclinical or clinical trials for renal fibrosis.	No significant toxicity has been reported; there is sufficient clinical safety data.	Lakshmanan et al. (2012)
DR8 (DHNNPQIR)	*In vivo*: 500 μg/kg/day, subcutaneous injection (s.c.), once daily, for 14 consecutive days (administration began 8 months after 5/6 nephrectomy). *In vitro*: 80–160 μM; after pretreatment, co-incubate C2C12 or HK-2 cells with TGF-β1 (5ng/mL) for 24–48 h.	A synthetic octapeptide derived from rapeseed protein. By downregulating CNR1 expression, it inhibits the phosphorylation and activation of the downstream ERK/ELK-1 signaling pathway, thereby blocking TGF-β1-induced fibrosis.	Indirect inhibition	Reduce collagen deposition in renal tissue; downregulate the expression of α-SMA, TGF-β1, collagen I, and fibronectin.	*In vivo*: C57BL/6J mice (CKD model induced by 5/6 nephrectomy). *In vitro*: C2C12 myoblasts, HK-2 cells (TGF-β1 stimulation).	Preclinical research (synthetic peptides; not yet in clinical trials).	No significant toxicity has been reported in the literature; CCK-8 assays showed no significant cytotoxicity toward C2C12 and HK-2 cells at concentrations of 80–160 μM.	Zhang et al. (2025a), Zhang et al. (2025b) and Zhang et al. (2025c)
Suplatast	*In vivo*: 100 mg/kg/day, dissolved in drinking water and administered ad libitum for 14 consecutive days (starting on postoperative day 1).	Th2 cytokine inhibitors suppress the production of Th2 cytokines such as IL-4, thereby inhibiting the phosphorylation and activation of ERK and STAT3, while simultaneously downregulating the mRNA expression of JAK1 and STAT3.	Indirect inhibition	Alleviates renal fibrosis: reduces collagen deposition and the area of fibrosis; decreases mRNA expression of KIM-1, TGF-β, and collagen I; improves renal function (lowers BUN and serum creatinine).	*In vivo*: Unilateral ureteral obstruction (UUO) model in male BALB/c mice (analyzed on day 14 post-surgery).	Approved drug (Japan, for bronchial asthma); currently in preclinical development for renal fibrosis.	These are drugs that are already on the market and have been supported by clinical safety data.	Honma et al. (2023)
N-Acetyl Cysteine (NAC)	*In vivo*: 84.8 mg/kg/day, dissolved in drinking water and administered ad libitum for 14 consecutive days (starting on postoperative day 1).	A classic ROS scavenger and glutathione precursor. It reduces oxidative stress by restoring glutathione peroxidase (GPx) activity suppressed by UUO, thereby indirectly inhibiting ERK1/2 phosphorylation (with no significant effect on JNK or p38).	Indirect inhibition	Reduce renal fibrosis: decrease collagen deposition and the area of fibrosis; lower mRNA expression of type III collagen and TNF-α; improve renal histopathological damage.	*In vivo*: Unilateral ureteral obstruction (UUO) model in male BALB/c mice (analyzed on day 14 post-surgery).	Approved drugs (for acetaminophen overdose, mucolytic therapy, *etc.*); currently in preclinical or drug repurposing studies for renal fibrosis.	As a drug that has been on the market for many years, it has a robust safety profile.	Honma et al. (2020)
TP0472993	*In vivo*: 0.3 or 3 mg/kg, administered by gavage twice daily for 12 consecutive days (FA model) or 7 consecutive days (UUO model).	A selective inhibitor of 20-hydroxytetradecenoic acid (20-HETE) synthesis that targets CYP4F2 and CYP4A11 (IC_5__0_ values of 37 nM and 425 nM, respectively). By reducing 20-HETE production, it inhibits the phosphorylation and activation of downstream ERK1/2 and STAT3 signaling pathways, thereby alleviating renal inflammatory responses.	Indirect inhibition	Reduction of renal fibrosis: decreased collagen deposition and collagen content (Sircol method); reduced levels of IL-1β and TNF-α in renal tissue; improvement in tubulointerstitial fibrosis.	*In vivo*: Male C57BL/6J mice (folic acid-induced nephropathy model, single intraperitoneal injection of 250 mg/kg folic acid, administered for 12 days; or UUO model, administered for 7 days post-surgery).	Preclinical studies (a novel selective inhibitor of 20-HETE synthesis; effective when administered orally).	At a concentration of 30 μM, it had no significant inhibitory effect on 55 types of receptors, enzymes, ion channels, and transporters; at therapeutic doses, it had no significant effect on mean arterial pressure, renal blood flow, or heart rate.	Hirata et al. (2023)
Erlotinib	*In vivo*: 80 mg/kg/day, administered by gavage once daily for 4 consecutive weeks, concurrently with adenine (0.2% w/w in the diet).	EGFR tyrosine kinase inhibitors (receptor tyrosine kinase inhibitors). By inhibiting EGFR phosphorylation, they block the activation of downstream ERK1/2 and STAT3 signaling pathways, while simultaneously reducing oxidative stress (MDA↓, GSH↑, CAT↑), upregulating the anti-apoptotic protein Bcl-2, and downregulating p53 expression.	Indirect inhibition	Reduce renal fibrosis: decrease collagen deposition and the area of fibrosis; lower TGF-β1 levels in renal tissue; improve tubular damage and inflammatory cell infiltration.	*In vivo*: Male CD-1 mice (a renal disease model induced by feeding a diet containing 0.2% adenine for 4 weeks).	Approved drugs (for non-small cell lung cancer and pancreatic cancer); currently in preclinical or drug repurposing studies for renal fibrosis.	Erlotinib may elevate serum urea levels, possibly due to its effect on the urea cycle in hepatocytes; liver transaminases should be monitored during clinical use. No significant nephrotoxicity was reported in this study.	Awad et al. (2020)
Eicosapentaenoic acid (EPA)	*In vivo*: 1 g/kg/day, intraperitoneal (i.p.) injection, once daily, for 8 consecutive weeks. *In vitro*: 30 μM, pretreated for 1 h, followed by co-incubation of mouse mesangial cells (MMCs) with PDGF (50 ng/mL) and/or high glucose (30 mM) for 24 h.	By inhibiting the phosphorylation of ERK1/2 and p38 MAPK (without affecting JNK or PI3K/Akt), it downregulates the expression and secretion of MCP-1, thereby reducing macrophage infiltration, inflammatory responses, and extracellular matrix deposition.	Indirect inhibition	Reduce mesangial matrix expansion and sclerosis; reduce the area of tubulointerstitial fibrosis; downregulate MCP-1 expression in renal tissue and F4/80-positive macrophage infiltration.	*In vivo*: KKAy/Ta type 2 diabetic mice (treatment initiated at 12 weeks of age, for 8 weeks); *In vitro*: mouse mesangial cells (MMCs), stimulated with PDGF (50 ng/mL) and/or high glucose (30 mM).	Already on the market/dietary supplement (for conditions such as hypertriglyceridemia); currently in preclinical or clinical trial phases for diabetic nephropathy.	According to the literature, LDH cytotoxicity assays have shown that EPA is non-cytotoxic at concentrations below 40 μM; no significant toxicity has been reported in animal studies.	Hagiwara et al. (2006)
Vorapaxar	*In vivo*: 15 or 30 mg/kg/day, administered by gavage once daily for 7 consecutive days (UUO) or 14 consecutive days (UIRI), with administration beginning immediately after surgery.	It blocks thrombin-induced PAR-1 activation, inhibits ERK1/2 MAPK phosphorylation, thereby downregulating the TGF-β/Smad3 signaling pathway, and alleviates inflammation (MCP-1, TNF-α), oxidative stress (Nox4, iNOS, ROS), and extracellular matrix deposition.	Indirect inhibition	Reduces collagen deposition; decreases mRNA and protein expression of fibronectin, collagen I, and α-SMA; downregulates KIM-1 expression; and reduces macrophage infiltration.	*In vivo*: BALB/c mice (UUO model, 7 days) and C57B6 mice (UIRI model, 14 days); *In vitro*: NRK-52E rat renal tubular epithelial cells (stimulated with 2 U/mL thrombin, 72 h).	Approved drugs (for reducing thrombotic cardiovascular events in patients with myocardial infarction or peripheral artery disease); currently in preclinical or drug repurposing studies for renal fibrosis.	No bleeding was observed in the mouse model (since mouse platelets lack PAR-1 and require PAR-3 for aggregation). While the risk of bleeding should be monitored in clinical practice, cardiovascular benefits have still been demonstrated in patients with impaired renal function.	Lok et al. (2020)
Camostat Mesilate (CM)	*In vivo*: 7 mg/day, subcutaneous implantation of sustained-release pellets, for 14 consecutive days. *In vitro*: 50–500 μM, 30-minute pretreatment, co-incubation with TGF-β1 (5 ng/mL) in NRK-49F cells.	Inhibits the phosphorylation of the TGF-β type I receptor (TβRI), blocks the downstream phosphorylation and activation of ERK1/2 and Smad2/3, and reduces the expression of pro-fibrotic factors (α-SMA, CTGF, PAI-1) and MMP-2.	Indirect inhibition	Reduces collagen deposition and hydroxyproline content; decreases mRNA expression of collagen I and III; downregulates levels of α-SMA, CTGF, proMMP-2, and MMP-2; and improves tubulointerstitial fibrosis.	*In vivo*: Male SD rats (UUO model, 14 days); *In vitro*: NRK-49F rat renal fibroblasts.	Approved drug (in Japan, for chronic pancreatitis, reflux esophagitis, etc.); currently in the preclinical/drug repurposing research phase for renal fibrosis.	As a marketed drug, CM has been supported by a body of clinical safety data.	Morinaga et al. (2013)

**Notes.**

MAPK1 is also known as ERK2; *in vivo*: *in vivo* experiments; *in vitro*: *in vitro* experiments; UUO: unilateral ureteral ligation model. Potential drug toxicity was referenced from drug labels and relevant literature. *In vivo* dilution method for qumetinib: Dissolve in DMSO and dilute to a final concentration of 0.5% Hyproelose (Sigma) and 2% Tween-80 (Sigma) in an aqueous solution mixture.

**Table 3:** Natural bioactive compounds targeting the MAPK1/ERK2 pathway.

Name	Source/Composition	Direction of action	Core mechanisms and downstream effects	Experimental Model/System	Evidence level	References
Vaccarin	Vaccariae	↓Inhibition	Inhibits phosphorylation at tyrosine residues Y845 and Y1173 of the epidermal growth factor receptor (EGFR) (Molecular Docking Validation), suppresses ERK1/2 activation, and alleviates epithelial-mesenchymal transition (EMT), inflammation, and oxidative stress.	High-fat diet (HFD)/streptozotocin (STZ)-induced T2DM mouse model	II	Gong et al. (2019) and Zhu et al. (2024)
Ganoderic acid	Ganoderma lucidum	↓Inhibition	Inhibits ERK phosphorylation while blocking the TGF-β/Smad pathway, reducing the expression of fibronectin (FN) and α-smooth muscle actin (α-SMA), and upregulating E-cadherin.	UUO mice	I	Geng et al. (2020)
Curcumin	Turmeric	↓Inhibition	Inhibits the MAPK/ERK signaling pathway, blocking Ang-II and TGF-β1-activated pro-fibrotic signal transduction	UUO mice, *In vitro* rat renal tubular epithelial cell line (NRK-52E)	I	Ni et al. (2014) and Sun et al. (2017)
Apocynin	The roots of Apocynum cannabinum (Canadian hemp) and Picrorhiza kurroa(Scrophulariaceae)	↓Inhibition	By targeting NOX2/NOX4, ROS production is reduced, thereby indirectly inhibiting ERK phosphorylation and the accumulation of myofibroblasts (DHE staining confirmed the reduction in ROS).	UUO rats	II	Cheng et al. (2016)
Tanshinone IIA	Salvia miltiorrhiza	↓Inhibition	Suppressed the increased levels of p-PERK, p-ELF2α, and ATF-4 proteins in diabetic rat kidney tissue, attenuated PERK signaling activity, modulated the TGF-β/inflammation axis, and reduced the expression of renal fibrosis-related markers.	SD rats; intraperitoneal injection of STZ (60 mg/kg) for 2 consecutive days; 2, 4, and 8 mg/kg daily, administered by injection for 6 weeks.	II	Chen et al. (2023) and Xu et al. (2020)
Quercetin	Widely found in fruits, vegetables, tea leaves, legumes, and wine, among others.	↓Inhibition	Targets EGFR, inhibits its phosphorylation and downstream ERK activation (as confirmed by Western blot analysis), and reduces podocyte apoptosis and proteinuria.	*In vitro* experiments: Conditionally immortalized mouse foot cells; *In vivo* experiments: Genetically diabetic C57BL/KSJ db/db mice	II	Chen et al. (2025) and Liu et al. (2021)
Astragaloside IV	Astragalus	↓Inhibition	Inhibits p-ERK and TGF-β-mediated α-SMA expression, thereby reducing fibrosis *via* the MAPK/NF-κB dual pathway (validated in vitro and *in vivo*).	Primary renal fibroblasts; UUO/DKD *in vivo* and *in vitro*	I	Che et al. (2015) and Xu et al. (2014)
Patchouli alcohol (PA)	Pogostemon cablin	↓Inhibition	Targets Ras/Raf-1, inhibits ERK1/2 phosphorylation (validated at both the mRNA and protein levels), and simultaneously downregulates the Renin/Ang II/TGF-β1 axis, thereby reducing fibrosis.	Spontaneously hypertensive rats (SHR), 8-week oral administration (20, 40, 80 mg/kg)	I	Li et al. (2023)
Osthole	Cnidium monnieri (L.) Cusson	↓Inhibition	Dual mechanism: (1) Inhibition of TGF-β/Smad transcription; (2) Direct targeting of the IL-11/ERK1/2 translational axis (validated *via* an IL-11-induced model and ERK inhibitors/activators), independent of TGF-β.	UUO mice; TGF-β1-induced HK-2 cells; IL-11-induced HK-2 cells (direct IL-11/ERK1/2 pathway validation)	I	Wu et al. (2021)
Pinocembrin (PIN)	Propolis and various plants (*e.g.*, Pinus species)	↓Inhibition	Targeted downregulation of CYP1B1 (*via* molecular docking and AAV9 overexpression recovery experiments) reduces ROS production, indirectly inhibits the MAPK pathway (ERK, p38, JNK), and alleviates inflammation and apoptosis.	*In vivo*: C57BL/6 mice (renal I/R injury; UUO-induced fibrosis); *In vitro*: HK-2 cells (H/R; TGF-β1 stimulation)	I	Zhang et al. (2025a) and Zhang et al. (2025b)
Alpha-mangostin (α-MG)	Garcinia mangostana L.	↓Inhibition	It selectively inhibits TGF-β1-induced ERK1/2 phosphorylation (without affecting p38 or JNK), blocks downstream Smad2/3 activation, and thereby inhibits EMT and fibrosis.	*In vivo*: C57BL/6 mice (UUO-induced renal fibrosis, 7 days; oral α-MG 10 or 20 mg/kg/day); *In vitro*: HK-2 cells (TGF-β1 20 ng/mL, 24 h; α-MG 2–4 μM)	I	Juan et al. (2023)
Fraxetin	Fraxinus rhynchophylla	↓Inhibition	Inhibits ERK1/2 phosphorylation (without affecting AKT), thereby inhibiting the progression of epithelial-mesenchymal transition (EMT). Reduces the expression of α-SMA, type I/IV collagen, fibronectin, and mesenchymal markers (N-cadherin, vimentin), while upregulating the expression of E-cadherin. Inhibits IS/TGF-β1-induced cell migration and extracellular matrix deposition. (Confirmed by a recovery assay using the MEK inhibitor U0126)	*In vivo*: C57BL/6 mice (UUO-induced renal fibrosis, 7 days; oral fraxetin 40 mg/kg/day); *In vitro*: MES13 cells (IS 50 μM), HK-2 cells (TGF-β1 10 ng/mL)	I	Hsieh et al. (2021)
Apigenin	Fruits, vegetables (*e.g.*, parsley, celery)	↓Inhibition	Activates AMPK phosphorylation while selectively inhibiting ERK1/2 phosphorylation (no effect on Smad2/3, p38, or JNK). Suppresses TGF-β1-induced renal fibroblast proliferation, fibroblast-to-myofibroblast differentiation (α-SMA), and ECM production (collagen I/III, CTGF, fibronectin). (Validation of the AMPK inhibitor Compound C and the ERK inhibitor LY3214996)	*In vitro*: NRK-49F rat renal fibroblasts (TGF-β1 2 ng/mL, 24 h; apigenin 5–20 μM)	I	Li et al. (2020)
Guaiacol	Angelica sinensis (Oliv.) Diels	↓Inhibition	Activates anti-fibrotic BMP/Smad1/5/9 signaling (upregulating p-Smad1/5/9) while suppressing pro-fibrotic ERK phosphorylation and Smad4 expression. Inhibits local RAAS (ACE) and NF-κB-mediated inflammation. Reduces ECM deposition (collagen, FN, α-SMA). (Computational pharmacology + molecular docking + AAV9 overexpression validation).	*In vivo*: Wistar rats (UUO, 21 days; oral guaiacol 15 or 30 mg/kg); In vitro: NRK-52E cells (rh-TGF-β1 10 ng/mL, 48 h; guaiacol 1 μM)	I	Kulkarni & Gaikwad (2026)
Vanillin	Vanilla planifolia	↓Inhibition	Suppresses ERK1/2 phosphorylation and downregulates TGF-β1/Smad2/3 signaling (molecular docking confirms binding to ALK5). Reduces pro-inflammatory cytokines (TNF-α, IL-6) and oxidative stress (MDA↓, GPx/CAT↑). Decreases α-SMA expression, collagen deposition, and Kim-1 expression.	*In vivo*: Wistar rats (thioacetamide 250 mg/kg, 6 weeks; vanillin 100 mg/kg i.p., early or late treatment)	I	Metwaly et al. (2022)
Puerarin	Pueraria lobata	↓Inhibition	Suppresses NOX4 expression, reducing oxidative stress (ROS) production. Inhibits MAPK signaling pathway activation (phosphorylation of ERK, p38, and JNK), thereby decreasing renal tubular epithelial cell apoptosis (caspase-3) and ECM deposition (fibronectin, collagen).	*In vivo*: C57BL/6 mice (UUO, 7 days; oral puerarin 50 or 100 mg/kg); *In vitro*: HK-2 cells (H_2_O_2_ 200 μM, 24 h; puerarin 50–100 μM)	I	Zhou et al. (2017)
CS-N	Cordyceps sinensis fermented powder; enriched in nucleosides/nucleobases (guanosine, uridine, adenosine, etc.)	↓Inhibition	Inhibits phosphorylation of p38 and ERK (no effect on JNK), thereby upregulating E-cadherin, downregulating α-SMA and ECM deposition (fibronectin, collagen I). (Confirmed by recovery experiments using the p38 inhibitor SB203580 and the ERK inhibitor U0126)	*In vivo*: C57BL/6 mice (STZ-induced diabetic nephropathy, 8 weeks; oral CS-N 40 or 80 mg/kg); *In vitro*: HK-2 cells (high glucose 30 mM, 48 h; CS-N 50–100 μg/mL)	I	Dong et al. (2019)
S-Methylmethionine (SMM)	Widely present in vegetables (cabbage, kale, garlic)	↓Inhibition	Suppresses ERK and NF-κB p65 phosphorylation, reducing pro-inflammatory cytokines (IL-1β, IL-6, TNF-α) and senescence-associated proteins (p16, p21, *γ*-H2A.X). Promotes macrophage M1-to-M2 polarization, restores phagocytic/migratory functions. (Validation of the ERK inhibitor FR180204 and the activator Ceramide C6)	*In vivo*: C57BL/6J mice (HFD + STZ-induced diabetic nephropathy, 10 weeks; oral SMM 50 or 100 mg/kg); *In vitro*: RAW264.7 macrophages (high glucose 33 mM, 24 h; SMM 2–4 mM)	I	Dong et al. (2026)
Higenamine	Aconitum species	↓Inhibition	Inhibits ASK1 phosphorylation, thereby suppressing downstream MAPK (ERK, p38) and NF-κB activation. Reduces TGF-β1, α-SMA, and collagen I expression, attenuating myofibroblast activation and ECM deposition. (*In vitro* and *in vivo* validation)	*In vivo*: SD rats (MI + 5/6 nephrectomy-induced CRS, 2 weeks; i.p. higenamine 0.5–4.5 mg/kg); *In vitro*: neonatal rat cardiac fibroblasts/myocytes (IS 10 μM or TGF-β1 10 ng/mL)	I	Deng et al. (2020)
Resveratrol	Widely found in grapes, berries, and peanuts	↓Inhibition	It activates AMPK, downregulates NOX4 expression, and reduces ROS production, thereby indirectly inhibiting ERK1/2 phosphorylation. It inhibits high-glucose-induced proliferation and activation of renal fibroblasts (α-SMA, FN) and alleviates renal interstitial fibrosis.	*In vivo*: db/db mice (a model of type 2 diabetic nephropathy; oral administration of 40 mg/kg/day for 12 weeks); *In vitro*: NRK-49F rat renal fibroblasts (30 mM glucose for 48 h)	I	He et al. (2016)
Cardamonin (CAD)	Alpinia katsumadai	↓Inhibition	Enhances the activity of antioxidant enzymes (SOD1, SOD2, CAT), reduces the production of ROS and MDA, thereby indirectly inhibiting the MAPK pathway (ERK, p38, JNK) and NF-κB phosphorylation. Inhibits inflammatory factors (TNF-α, IL-6, IL-1β) and apoptosis (BAX, cleaved caspase-3), thereby alleviating tubular damage and ECM deposition (collagen I/III, FN, α-SMA).	*In vivo*: C57BL/6 mice (renal ischemia-reperfusion injury; UUO-induced renal fibrosis, oral administration of 100 mg/kg/day); *In vitro*: HK-2 cells (hepatocyte-like cells; TGF-β1 5 ng/mL)	I	Zhang et al. (2023)
trans-THSG	Polygonum multiflorum	↓Inhibition	Inhibits TGF-β-induced phosphorylation of ERK1/2 and Smad1/2, reduces the release of pro-inflammatory cytokines (IL-17, TNF-α, TGF-β), alleviates oxidative stress (increased SOD and GSH, decreased MDA), downregulates the expression of fibrosis markers (α-SMA, desmin) and MMP2/MMP9, and mitigates collagen deposition and renal injury.	*In vivo*: SD rats (CCl_4_-induced model of liver fibrosis and kidney injury; oral administration of 100 or 300 mg/kg/day for 8 weeks)	I	Long et al. (2019)
Nimbidiol	Azadirachta indica	↓Inhibition	By lowering blood glucose levels, it inhibits the phosphorylation of the TGF-β1/Smad and MAPK (ERK1/2, p38, JNK) signaling pathways. This reduces M1 macrophage infiltration and inflammatory cytokines, inhibits epithelial-mesenchymal transition (EMT), decreases extracellular matrix (ECM) deposition, and improves renal function.	*In vivo*: C57BL/6-Ins2Akita/J type 1 diabetic mice (administered *via* subcutaneous microinfusion pump at 0.40 mg/kg/day for 8 weeks)	I	Juin et al. (2022)
Melittin	Bee venom (Apis mellifera)	↓Inhibition	Inhibits TGFβRII phosphorylation, thereby simultaneously blocking the phosphorylation and activation of downstream Smad2/3, ERK1/2, and JNK (with no effect on p38 and PI3K/Akt). Validation using the ERK inhibitor PD98059 and the JNK inhibitor SP600125 confirmed that it downregulates the expression of PAI-1, type I collagen, and fibronectin by inhibiting the ERK/JNK pathway.	*In vitro*: NRK-49F rat renal fibroblasts (TGF-β 5 ng/mL, 10 h; melittin 0.1–1 μg/mL)	I	Park et al. (2014)
Ginsenoside Rg1	Panax notoginseng	↓Inhibition	Inhibits ERK1/2 phosphorylation and blocks TGF-β1-induced epithelial-to-myofibroblastic transdifferentiation (EMT). It upregulates the epithelial marker E-cadherin, downregulates the myofibroblastic marker α-SMA, and reduces the synthesis and secretion of extracellular matrix components (collagen I and fibronectin).	*In vitro*: NRK-52E rat renal tubular epithelial cells (TGF-β1 10 ng/mL, 72 h; ginsenoside Rg1 10–40 ng/mL)	I	Xie et al. (2009)
Allicin	Allium sativum	↓Inhibition	Inhibits TGF-β1 expression, reduces the p-ERK1/2/total ERK1/2 ratio, decreases collagen I deposition and ECM accumulation, alleviates glomerular basement membrane thickening and tubulointerstitial fibrosis, and improves renal function and lipid metabolism.	*In vivo*: SD rats (STZ-induced diabetic nephropathy model; administered orally at doses of 15, 30, or 45 mg/kg/day for 12 weeks)	I	Huang et al. (2017)

**Notes.**

MAPK1 is also known as ERK2; ↑ indicates upregulation/activation, ↓ indicates downregulation/inhibition. Evidence grades are categorized into three levels based on whether they directly impact the ERK pathway: Level I indicates direct evidence/pathway for MAPK1/ERK2 inhibition; Level II indicates sufficient anti-fibrotic/anti-inflammatory effects of the drug, but regulation of MAPK1/ERK2 is inferred/indirect; Level III indicates systematic reviews/meta-analyses supporting renal protection, but evidence for MAPK1/ERK2 regulation is weak.)

### Inhibition of the MAPK1/ERK2 pathway by synthetic drugs

Abnormal activation of the MAPK1/ERK2 signaling pathway is closely linked to the initiation and progression of renal fibrosis. Consequently, inhibiting this pathway has emerged as a key therapeutic strategy in CKD. Several synthetic drugs have demonstrated potential in preclinical studies to delay renal fibrosis by modulating MAPK1/ERK2 signaling. Table 2 summarizes the mechanisms of action, experimental models, and anti-fibrotic effects of these agents, summarizing the current status of research in this area (Andrikopoulos et al., 2019; Awad et al., 2020; Bhadange & Gaikwad, 2025; Chen et al., 2016; Chen et al., 2026; Cheng et al., 2021; Choi et al., 2018; Feng et al., 2023; Hagiwara et al., 2006; Han et al., 2018; Hirata et al., 2023; Honma et al., 2024; Honma et al., 2023; Honma et al., 2020; Juin et al., 2022; Lakshmanan et al., 2012; Li et al., 2018; Li et al., 2017; Liu et al., 2015; Liu et al., 2026; Lok et al., 2020; Morinaga et al., 2013; Nady, Abd El-Raouf & El-Sayed, 2024; Qin et al., 2015; Shen et al., 2016; Sun et al., 2023; Zhang et al., 2025b).

In summary, drugs such as linagliptin, vildagliptin, and anlotinib have shown promising results in targeting renal fibrosis *via* inhibition of MAPK1/ERK2 signaling. However, most synthetic agents specifically designed to inhibit this pathway in renal fibrosis remain in early research and development phases. Given the absence of approved therapeutic agents explicitly targeting renal fibrosis, future studies should focus on elucidating interactions between MAPK1/ERK2 and other signaling pathways, such as the PI3K/Akt pathway. This includes the exploration of combination therapies and the development of more selective and safer MAPK1/ERK2 inhibitors. Such approaches may offer new strategies for advancing dedicated anti-fibrotic therapies for renal fibrosis.

### Inhibition of the MAPK1/ERK2 pathway by natural bioactive compounds

Natural bioactive compounds derived from traditional medicinal herbs represent a rich source of candidate molecules capable of modulating the MAPK1/ERK2 pathway. Unlike complex multi-component herbal formulations, these structurally defined single molecules allow for a clear attribution of anti-fibrotic effects to pathway inhibition, thereby facilitating mechanistic clarity. Table 3 details the mechanisms through which natural bioactive compounds identified by our systematic search target the MAPK1/ERK2 pathway, laying a foundation for future drug development (Che et al., 2015; Chen et al., 2025; Chen et al., 2023; Cheng et al., 2016; Deng et al., 2020; Dong et al., 2026; Dong et al., 2019; Geng et al., 2020; Gong et al., 2019; He et al., 2016; Hsieh et al., 2021; Huang et al., 2017; Juan et al., 2023; Juin et al., 2022; Kulkarni & Gaikwad, 2026; Li et al., 2023; Li et al., 2020; Liu et al., 2021; Long et al., 2019; Metwaly et al., 2022; Ni et al., 2014; Park et al., 2014; Sun et al., 2017; Wu et al., 2021; Xie et al., 2009; Xu et al., 2020; Xu et al., 2014; Zhang et al., 2023; Zhang et al., 2025a; Zhang et al., 2025b; Zhou et al., 2017; Zhu et al., 2024).

Although these natural bioactive compounds exhibit significant therapeutic potential, their clinical advancement is hindered by an incomplete understanding of their complex *in vivo* pharmacological behavior. Future research should shift from isolated pharmacodynamic validation toward integrated pharmacodynamic-pharmacokinetic (PD-PK) network analyses. Such an integrated approach would elucidate the multi-target mechanisms and *in vivo* behaviors of these compounds, providing a robust theoretical basis for rational combination therapy design, integrating natural bioactive agents with synthetic drugs.

Beyond the compounds summarized in Tables 2 and 3, some studies have also identified biologic agents capable of modulating the MAPK1/ERK2 pathway in renal fibrosis (Kalina et al., 2025). For instance, Kalina et al. (2025) demonstrated that targeting coagulation factor XIIa (FXIIa) using the monoclonal antibody 3F7 produced significant anti-fibrotic effects in UUO mice. Mechanistically, FXIIa activates ERK1/2 signaling through the uPAR-β1 integrin-EGFR complex. Treatment with 3F7 significantly reduced phosphorylation of EGFR, Akt, and ERK1/2 in kidney tissue, and abolished FXIIa-induced ERK1/2 activation in renal epithelial cells *in vitro*. Functionally, 3F7 attenuated tubulointerstitial fibrosis, as indicated by decreased collagen I, fibronectin, and α-SMA deposition, reduced apoptosis of tubular epithelial cells, and improved renal histological scores. Although 3F7 remains at the preclinical stage for renal fibrosis, its target, FXIIa, has already been clinically validated (garadacimab, approved for hereditary angioedema), with no notable toxicity reported (Craig et al., 2023; Drulyte et al., 2024; Fung, 2025). This evidence highlights the therapeutic potential of targeting the FXIIa–ERK1/2 axis, expanding the therapeutic scope for MAPK1/ERK2 in renal fibrosis (Kalina et al., 2025). Similarly, recombinant human ACE2 (rhACE2) has exhibited anti-fibrotic effects. Chen et al. (2016) showed that rhACE2 alleviated renal fibrosis in ApoE-deficient mice by degrading Ang II and generating Ang-(1-7), activating the Mas receptor and indirectly suppressing ERK1/2 phosphorylation. The specificity of this signaling mechanism was confirmed using the Mas receptor antagonist A779 (Chen et al., 2016). Furthermore, emerging evidence identifies intracellular adaptor proteins as critical regulators of ERK activation. Yu et al. (2022) reported that LIM and cysteine-rich domains 1 (LMCD1) acts as a novel driver of renal fibrosis, with significantly increased expression in obstructed kidneys and TGF-β1-treated tubular epithelial cells. LMCD1 deficiency reduced ERK1/2 phosphorylation, decreased fibrotic marker expression and tubular cell apoptosis, and mitigated UUO-induced renal injury (Yu et al., 2022). This positions LMCD1 as a promising therapeutic target and upstream regulator of ERK signaling in renal fibrosis. In addition, studies have shown that renalase, a secreted flavoprotein significantly downregulated in obstructed kidneys, effectively reduces renal interstitial fibrosis when overexpressed *via* adenoviral vectors in UUO rats (Wu et al., 2017). Mechanistically, renalase inhibits TGF-β1-induced EMT by suppressing ERK1/2 phosphorylation. This protective effect is abolished by ERK1 overexpression, confirming renalase as an upstream regulator of ERK-mediated fibrotic signaling (Wu et al., 2017). Collectively, these findings highlight the crucial regulatory role of the ERK signaling pathway at multiple levels, including intracellular adaptor proteins and secreted signaling molecules, in renal fibrosis pathogenesis.

## Discussion

Renal fibrosis, a common pathological feature of CKD, significantly contributes to its recurrent progression and chronicity. Without effective intervention, renal fibrosis ultimately leads to renal failure and death. Identifying specific therapeutic targets to delay fibrosis thus holds considerable promise for developing novel clinical treatment strategies for CKD.

MAPK1/ERK2, a central component of the ERK signaling pathway, is activated by diverse stimuli, including TGF-β1, hyperglycemia, lipotoxicity, oxidative stress, and inflammatory factors. Its activation mediates mitochondrial-associated membrane (MAM) disruption and mitochondrial dysfunction, regulates inflammatory responses, and induces phenotypic changes in renal tubular epithelial cells, collectively accelerating renal fibrosis progression. Although research into MAPK1/ERK2 signaling in renal fibrosis remains at an early stage, accumulating studies indicate that specific synthetic drugs and natural bioactive compounds targeting this pathway can ameliorate fibrotic outcomes.

The evolving concept of pEMT further clarifies the significance of MAPK1/ERK2 signaling in renal fibrosis. The recognized role of ERK in regulating cell cycle progression, transcription factor activity, and secretory functions aligns closely with the characteristics of pEMT (Huang, Fu & Ma, 2023b; Sheng & Zhuang, 2020). ERK-induced expression of Snail1 and Twist not only promotes mesenchymal marker expression but also triggers cell-cycle arrest at the G2/M phase and the secretion of profibrotic factors by renal tubular epithelial cells (Huang et al., 2023a; Lovisa et al., 2015). These findings imply that the therapeutic benefits of MAPK1/ERK2 inhibition may partly derive from reversing pEMT, thus restoring epithelial homeostasis and interrupting profibrotic paracrine signaling. Recent studies have demonstrated that anti-platelet therapy attenuates pEMT and fibrosis by interfering with TGF-β1 signaling further supports epithelial plasticity as a therapeutic target for renal fibrosis (Rustiasari et al., 2025). Future research combining lineage tracing with single-cell analyses could determine whether MAPK1/ERK2 inhibitors specifically target and reverse pEMT in distinct epithelial subpopulations (Sheng & Zhuang, 2020).

Although targeting MAPK1/ERK2 signaling holds substantial promise, clinical translation faces several challenges. Regarding target specificity, ERK1 and ERK2 exhibit high sequence homology and share similar upstream activation mechanisms and downstream substrates (Fu et al., 2022). However, significant differences exist in their expression levels in renal cells, ERK2 expression is substantially higher, contributing the majority of total ERK activity. Most studies have analyzed ERK1/2 as a single entity, generally viewing these isoforms as functionally redundant under normal physiological conditions (Buscà et al., 2015; Buscà, Pouysségur & Lenormand, 2016; Frémin et al., 2015). Nevertheless, given ERK2′s predominant contribution to total ERK activity, specifically targeting ERK2 markedly reduces overall ERK activity. This makes ERK2 essential in pathological contexts such as renal fibrosis, where total ERK activity thresholds critically determine outcomes. The fundamental difference between ERK1 and ERK2 is thus quantitative, based on expression levels, rather than qualitative differences in kinase function.

Further clarification of ERK1 and ERK2 distinctions is necessary for understanding ERK2′s specific role in renal fibrosis. Structurally, ERK1 contains an additional 17-amino acid N-terminal extension, resulting in slower nucleocytoplasmic shuttling compared to ERK2, potentially reducing ERK1′s nuclear signaling efficiency (Marchi et al., 2008). Furthermore, within the so-called “dimerization domain,” the PE/DHD sequence differs between the two isoforms: ERK1 carries PEHD, whereas ERK2 carries PDHD. Although this conservative amino acid substitution does not alter the overall charge, the slightly bulkier side chain of glutamic acid in ERK1 may introduce steric hindrance that affects binding to specific interacting proteins (Buscà, Pouysségur & Lenormand, 2016). Despite these structural differences, the fundamental distinction between ERK1 and ERK2 does not lie in qualitative differences in catalytic function; both isoforms share highly conserved kinase domains, identical upstream activation mechanisms, and largely overlapping substrate profiles (Buscà, Pouysségur & Lenormand, 2016; Eblen, 2018; Von Kriegsheim et al., 2009). Rather, the key difference resides in the quantitative regulation of expression: in most mammalian cells, ERK2 is expressed at substantially higher levels than ERK1, accounting for approximately 80% of the total ERK protein pool (Lefloch, Pouysségur & Lenormand, 2008). This disparity is attributed to stronger proximal promoter activity, a larger gene structure, and more complex post-transcriptional regulation associated with the longer 3′UTR of the erk2 gene (Buscà, Pouysségur & Lenormand, 2016).

Based on these observations, the relationship between ERK2 and the broader ERK1/2 system requires reconsideration. Accumulating evidence indicates that many biological effects previously attributed to “ERK2-specific functions” correlate with the extent of reduction in total ERK activity, regardless of which isoform is targeted experimentally (Erdenebaatar et al., 2023; Frémin et al., 2015; Lefloch, Pouysségur & Lenormand, 2008). The more pronounced phenotypes observed following ERK2 knockdown reflect its greater contribution to total ERK activity, rather than unique substrates or signaling outputs absent in ERK1. This interpretation is supported by gene replacement studies demonstrating that systemic overexpression of ERK1 can compensate for the loss of ERK2, restoring normal mouse development and viability (Frémin et al., 2015). Thus, in the context of renal fibrosis, ERK2 should be regarded as the primary contributor to total ERK activity rather than a functionally irreplaceable kinase isoform. The “central role of ERK2” thus reflects its dominant quantitative contribution: owing to its higher expression, ERK2 serves as the major source of ERK activity in renal cells, and its inhibition most effectively reduces total ERK signaling. These quantitative differences are further amplified under pathological conditions, conferring ERK2 with the status of a functionally dominant isoform.

From this perspective, the rationale for developing highly selective MAPK1/ERK2 inhibitors is not to target a functionally unique isoform, but to achieve precise therapeutic modulation by preferentially reducing the major contributor to total ERK activity while minimizing disruption to ERK1. This approach represents a promising direction for precision drug development in renal fibrosis, with the potential to improve efficacy while limiting off-target effects. The identification of reliable biomarkers is essential for clinical translation. In addition to phosphorylated p-ERK1/2 levels (Yan et al., 2024), assessment of downstream effectors such as phosphorylated RSK, combined with analysis of MAPK/ERK-related signaling molecules in urinary exosomes, may provide a clinically feasible and dynamic readout of pathway activity in the kidney. Such measures could offer valuable evidence for evaluating the therapeutic efficacy of targeted interventions (Cao et al., 2025). Future research should prioritize validation of the specificity and sensitivity of these biomarkers. The clinical development of MAPK1/ERK2 inhibitors requires a clearly defined translational pathway. A major challenge lies in the toxicity profiles of current drugs. Therefore, comprehensive safety evaluation should follow a stepwise approach, progressing from *in vitro* studies and UUO animal models to CKD models in large animals, such as non-human primates, before advancing to early-phase clinical trials. Concurrently, systematic investigation of the synergistic effects between natural bioactive compounds and synthetic drugs may provide critical evidence for developing novel therapeutic strategies with well-defined mechanisms, improved efficacy, and reduced toxicity.

The role of MAPK1/ERK2 signaling remains incompletely understood in fibroblasts, macrophages, and other renal cell types. Two significant barriers also hinder the clinical translation of MAPK1/ERK2 inhibitors: ensuring sufficient selectivity and safety. Thus, the development of chemically synthesized drugs with high specificity toward this pathway remains an active research priority. A notable challenge in kinase-targeted therapies, including those targeting MAPK1/ERK2, is compensatory activation of alternative signaling pathways. Preclinical evidence indicates that pharmacological inhibition of ERK signaling can trigger counter-regulatory activation of parallel cascades, potentially limiting therapeutic efficacy (Nutter, Haylor & Khwaja, 2015). For instance, in a rat model of progressive renal fibrosis induced by 5/6 nephrectomy (SNx), treatment with the MEK inhibitor CI-1040 cabolished renal ERK1/2 phosphorylation throughout the treatment duration. However, this effective ERK blockade failed to ameliorate proteinuria, renal tissue fibrosis, or other manifestations of declining renal function. Mechanistic analyses revealed that ERK1/2 inhibition induced compensatory upregulation of p38 and JNK MAP kinases, along with increased expression of plasminogen activator inhibitor-1 (PAI-1), a key inhibitor of matrix metalloproteinases (Nutter, Haylor & Khwaja, 2015). This finding clearly demonstrates that selective ERK pathway inhibition can result in diminished therapeutic efficacy or treatment failure due to compensatory activation of alternative MAPK pathways, highlighting the importance of understanding signaling crosstalk in renal fibrosis therapy.

In addition to p38 and JNK signaling, the PI3K/AKT pathway represents another compensatory route. Studies have shown that TGF-β1, a central pro-fibrotic cytokine, simultaneously activates ERK1/2, p38 MAPK, and PI3K/AKT pathways, driving EMT and ECM production (Huang, Fu & Ma, 2023b; Lu et al., 2024). Specifically, in renal tubular epithelial cells, TGF-β1 downregulates the protective scaffold protein PDZ domain-containing 1 (PDZK1) through p38 MAPK and PI3K/AKT pathways independent of ERK/JNK signaling, thereby enhancing oxidative stress and fibrosis (Lu et al., 2024). Consequently, even effective ERK1/2 inhibition may not fully suppress fibrosis, as parallel signaling *via* p38 or PI3K/AKT continues to promote profibrotic programs. Conversely, pharmacological inhibition of p38/JNK or PI3K/AKT signaling restores PDZK1 expression and mitigates renal fibrosis in murine CKD models (Lu et al., 2024), suggesting potential therapeutic synergy from combined pathway targeting.

The ERK5 signaling pathway introduces additional complexity. As an alternative MAPK cascade responsive to mitogenic and stress signals, ERK5 shares functional overlap with ERK1/2 in regulating cell proliferation and survival (Nishimoto & Nishida, 2006; Xiao et al., 2025). In oncology, resistance to selective ERK1/2 inhibitors has been attributed to compensatory ERK5 activation, prompting the development of dual ERK1/2–ERK5 inhibitors such as SKLB-D18. Compared with selective inhibitors, these dual-target agents exhibit enhanced antitumor efficacy in preclinical models (Wang et al., 2020; Xiao et al., 2025). Although the specific role of ERK5 in renal fibrosis remains unclear, potential compensatory activation following ERK2 inhibition warrants careful investigation.

Collectively, these findings highlight a critical consideration for MAPK1/ERK2-targeted therapy in renal fibrosis: therapeutic success depends not on blocking a single kinase but rather on the overall impact on the integrated signaling network. Compensatory upregulation of p38, JNK, PI3K/AKT, or potentially ERK5 may circumvent ERK2 inhibition, perpetuating fibrotic progression. Rational therapeutic strategies should therefore encompass: (i) combination approaches simultaneously targeting multiple profibrotic pathways (*e.g.*, concurrent ERK and p38 or PI3K/AKT inhibition); (ii) development and clinical testing of dual-specificity inhibitors targeting both ERK1/2 and ERK5 (*e.g.*, SKLB-D18); and (iii) optimization of dosing regimens (such as sequential or intermittent administration) to minimize compensatory feedback activation. Detailed characterization of signaling crosstalk mechanisms in specific renal cell types will be essential for designing effective, durable anti-fibrotic therapies.

The identification of autophagy as an upstream regulator of MAPK1/ERK2 signaling opens new avenues for combination therapy in renal fibrosis. Livingston et al. (2024) demonstrated that during maladaptive renal repair, autophagy promotes fibroblast activation through the MAPK1/ERK2-EGR1-FGF2 signaling axis (Livingston et al., 2024). Interestingly, this autophagy-mediated mechanism intersects with the mitochondria-associated membrane (MAM)-mitochondrial dysfunction pathway (Liu et al., 2024). Autophagy acts upstream to activate ERK, while ERK-driven disruption of MAM integrity generates mitochondrial ROS that can further amplify autophagy (Liu et al., 2024; Livingston et al., 2024). This reciprocal interaction establishes a positive feedback loop, autophagy activating ERK, and ERK-mediated mitochondrial dysfunction potentiating autophagy, that may sustain and propagate fibrotic signaling within injured tubular cells. This reciprocal relationship suggests that dual inhibition of autophagy and MAPK1/ERK2 could provide synergistic antifibrotic effects. For instance, combining autophagy inhibitors (such as hydroxychloroquine) with ERK1/2 inhibitors (such as VX-11e) could concurrently disrupt upstream autophagy-mediated ERK activation and downstream ERK-induced fibrotic signaling. Notably, emerging evidence suggests direct interactions between hydroxychloroquine and ERK signaling (El-Maadawy et al., 2025). In skeletal muscle fibrosis models, hydroxychloroquine treatment ameliorates fibrosis by inhibiting ERK1/2 phosphorylation (Edwards et al., 2020). However, the regulatory effect of hydroxychloroquine on ERK signaling may vary by cell type. For example, Sung et al. (2025) reported that hydroxychloroquine increased ERK phosphorylation while suppressing autophagy in trophoblast cells (Sung et al., 2025). Despite context-specific variability, current evidence supports hydroxychloroquine’s ability to modulate ERK signaling alongside autophagy inhibition (El-Maadawy et al., 2025; Sung et al., 2025). Therefore, systematically evaluating this dual-blockade strategy in preclinical CKD models represents a promising therapeutic approach.

Therefore, identifying effective combination therapies remains a highly promising research direction for renal fibrosis. Considering the compensatory signaling mechanisms discussed above, rational strategies should incorporate dual inhibition of MAPK1/ERK2 alongside p38 MAPK or PI3K/AKT pathways, dual targeting of ERK1/2 and ERK5 using agents such as SKLB-D18 (Xiao et al., 2025); or combining ERK inhibition with autophagy modulators. Crucially, successful clinical translation will depend on establishing a robust safety framework, involving low-dose, short-duration regimens with continuous monitoring of renal function in real-time.

Due to the complexity of signaling networks and compensatory mechanisms, combination therapy remains the most promising approach in renal fibrosis research. Effective strategies include dual targeting of MAPK1/ERK2 and ERK5, or combining ERK inhibition with autophagy inhibitors. A key requirement for successful clinical translation is a robust safety profile based on low-dose, short-course treatments and continuous renal function monitoring.

As a narrative review, this article inherently involves some degree of subjectivity in study selection and qualitative interpretation, and thus does not provide a quantitative synthesis of data. Nonetheless, by integrating current literature, it offers a valuable and focused mechanistic overview of the MAPK1/ERK2 signaling pathway in renal fibrosis, providing a conceptual foundation for future investigations.

## Conclusion

This review has deliberately emphasized synthetic drugs and natural bioactive compounds to provide a clear mechanistic understanding of therapeutic interventions targeting the MAPK1/ERK2 pathway. This approach facilitates a more precise interpretation of the causal relationship between pathway inhibition and observed anti-fibrotic effects. Importantly, while ERK1 and ERK2 are functionally redundant at the individual molecule level, ERK2 dominates overall ERK activity in most renal cell types due to its substantially higher expression, positioning it as the primary driver of fibrotic signaling. Furthermore, adopting the concept of pEMT provides deeper insight into how MAPK1/ERK2 signaling regulates cellular plasticity during fibrosis, and offers refined endpoints for evaluating therapeutic outcomes (Huang, Fu & Ma, 2023b; Sheng & Zhuang, 2020). Future studies involving complex herbal formulations would benefit significantly from advanced analytical methods, such as integrated pharmacodynamic–pharmacokinetic (PD-PK) network analyses, to decipher their multi-component, multi-target actions and clearly identify the roles of active constituents.

Overall, the MAPK1/ERK2 signaling pathway, particularly through ERK2 as the dominant isoform in renal cells, holds considerable therapeutic promise for renal fibrosis, despite several challenges. Continued research is expected to further validate targeting this pathway as a viable strategy for alleviating renal fibrosis and managing CKD.

##  Supplemental Information

10.7717/peerj.21529/supp-1 Supplemental Information 1 Full search strings for all databases

10.7717/peerj.21529/supp-2 Supplemental Information 2 Chemical structures and CAS numbers of synthetic drugs and natural bioactive compounds targeting the MAPK1/ERK2 pathway

## Additional Information and Declarations

### Data Availability

The following information was supplied regarding data availability: This is a literature review.
