# Supplementary material for: From mechanism to therapeutics: targeting the mitogen-activated protein kinase 1 (MAPK1)/extracellular signal-regulated kinase 2 (ERK2) pathway in renal fibrosis
Source: PeerJ. 2026 Jul 21;14:e21529. doi: 10.7717/peerj.21529 (PMC13398395; doi:10.7717/peerj.21529)
Supplement: Supplemental Information 1 [file peerj-14-21529-s001.docx]

**Supplementary Table S1. Full search strings for all databases**

| Database | Phase 1 Broad search for MAPK1/ERK2 in renal fibrosis | Phase 2 Targeted search for therapeutic interventions |
| --- | --- | --- |
| PubMed | ("erk1/2" OR "extracellular signal-regulated kinase 1/2" OR "MAPK3/MAPK1" OR "mitogen-activated protein kinase 3/1" OR "MAPK1" OR "ERK2") AND ("renal fibrosis" OR "renal fibrosis" OR "kidney scarring" OR "nephrogenic fibrosis" OR "renal interstitial fibrosis") | ("renal fibrosis" OR "kidney fibrosis" OR "diabetic nephropathy" OR "diabetic kidney disease" OR "chronic kidney disease" OR "tubulointerstitial fibrosis") AND ("MAPK1" OR "ERK2" OR "extracellular signal-regulated kinase 2" OR "mitogen-activated protein kinase 1" OR "MAPK/ERK pathway" OR "ERK1/2" OR "p-ERK") AND ("inhibitors" OR "antagonists" OR "drugs" OR "pharmacological" OR "natural products" OR "traditional Chinese medicine" OR "bioactive compounds" OR "flavonoid" OR "terpenoid" OR "saponin") |
| Web of Science | TS=("erk1/2" OR "extracellular signal-regulated kinase 1/2" OR "MAPK3/MAPK1" OR "mitogen-activated protein kinase 3/1" OR "MAPK1" OR "ERK2") AND TS=("renal fibrosis" OR "kidney scarring" OR "nephrogenic fibrosis" OR "renal interstitial fibrosis") | TS=("renal fibrosis" OR "kidney fibrosis" OR "diabetic nephropathy" OR "diabetic kidney disease" OR "chronic kidney disease" OR "tubulointerstitial fibrosis") AND TS=("MAPK1" OR "ERK2" OR "extracellular signal-regulated kinase 2" OR "mitogen-activated protein kinase 1" OR "MAPK/ERK pathway" OR "ERK1/2" OR "p-ERK") AND TS=("inhibitors" OR "antagonists" OR "drugs" OR "pharmacological" OR "natural products" OR "traditional Chinese medicine" OR "bioactive compounds" OR "flavonoid" OR "terpenoid" OR "saponin") |
| CNKI | (SU='erk1/2' OR SU='细胞外信号调节激酶1/2' OR SU='MAPK3/MAPK1' OR SU='丝裂原活化蛋白激酶3/1' OR SU='MAPK1' OR SU='ERK2') AND (SU='肾纤维化' OR SU='肾脏纤维化' OR SU='肾间质纤维化')  (English translation: ERK1/2, extracellular signal-regulated kinase 1/2, MAPK3/MAPK1, mitogen-activated protein kinase 3/1, MAPK1, ERK2; renal fibrosis, kidney fibrosis, renal interstitial fibrosis) | (SU='肾纤维化' OR SU='肾脏纤维化' OR SU='糖尿病肾病' OR SU='慢性肾脏病' OR SU='肾间质纤维化') AND (SU='MAPK1' OR SU='ERK2' OR SU='细胞外信号调节激酶2' OR SU='丝裂原活化蛋白激酶1' OR SU='MAPK/ERK信号通路' OR SU='ERK1/2' OR SU='p-ERK') AND (SU='抑制剂' OR SU='拮抗剂' OR SU='药物' OR SU='药理学' OR SU='天然产物' OR SU='中药' OR SU='生物活性成分' OR SU='黄酮' OR SU='萜类' OR SU='皂苷')  (English translation: renal fibrosis, diabetic nephropathy, chronic kidney disease, tubulointerstitial fibrosis; MAPK1, ERK2, extracellular signal-regulated kinase 2, mitogen-activated protein kinase 1, MAPK/ERK pathway, ERK1/2, p-ERK; inhibitors, antagonists, drugs, pharmacological, natural products, traditional Chinese medicine, bioactive compounds, flavonoid, terpenoid, saponin) |
