# Supplementary material for: From mechanism to therapeutics: targeting the mitogen-activated protein kinase 1 (MAPK1)/extracellular signal-regulated kinase 2 (ERK2) pathway in renal fibrosis
Source: PeerJ. 2026 Jul 21;14:e21529. doi: 10.7717/peerj.21529 (PMC13398395; doi:10.7717/peerj.21529)
Supplement: Supplemental Information 2 [file peerj-14-21529-s002.docx]

**Supplementary Table S2. Chemical structures and CAS numbers of synthetic drugs and natural bioactive compounds targeting the MAPK1/ERK2 pathway**

| Category | Compound Name | Chemical Structure | CAS Registry Number |
| --- | --- | --- | --- |
| Synthetic Drug | Linagliptin | C_25_H_28_N_8_O_2_ | 668270-12-0 |
|  | Vildagliptin | C_17_H_25_N_3_O_2_ | 274901-16-5 |
|  | Pirfenidone | C_12_H_11_NO | 53179-13-8 |
|  | Trametinib | C_26_H_23_FIN_5_O_4_ | 871700-17-3 |
|  | Erlotinib | C_22_H_23_N_3_O_4_ | 183321-74-6 |
|  | Aranotin | C_20_H_18_N_2_O_7_S_2_ | 19885-51-9 |
|  | CaMKII Inhibitory Peptide AIP | C_78_H_142_N_22_O_20_ . C_2_HF_3_O_2_ | 167114-91-2 |
|  | Fluorofenidone | C_12_H_10_FNO | 848353-85-5 |
|  | Urolithin A | C_13_H_8_O_4_ | 1143-70-0 |
|  | Risedronate (RIS) | C_7_H_11_NO_7_P_2_ | 105462-24-6 |
|  | Metformin | C_4_H_11_N_5_ | 657-24-9 |
|  | Erythropoietin (EPO) | C_134_H_226_N_38_O_41_ | 11096-26-7 |
|  | SHP099 (SHP2 inhibitor) | C_16_H_19_Cl_2_N_5_ | 1801747-42-1 |
|  | Mefunidone (MFD) | C_21_H_26_ClF_3_N_4_O_2_ | N/A |
|  | Olmesartan  DR8 (DHNNPQIR) | C_24_H_26_N_6_O_3_  C₄₀H₆₄N₁₆O₁₄ (octapeptide, sequence: Asp-His-Asn-Asn-Pro-Gln-Arg) | 144689-24-7  N/A |
|  | Suplatast | C_16_H_26_NO_4_S | 94055-75-1 |
|  | N-Acetyl Cysteine (NAC) | C_5_H_9_NO_3_S | 616-91-1 |
|  | CG200745 | C_24_H_33_N_3_O_4_ | 936221-33-9 |
|  | TP0472993 | C_16_H_20_N_4_O_2_ | 2126874-77-7 |
|  | Erlotinib | C_22_H_23_N_3_O_4_ | 183321-74-6 |
|  | Eicosapentaenoic acid (EPA) | C_20_H_30_O_2_ | 32839-30-8 |
|  | Vorapaxar | C_29_H_33_FN_2_O_4_ | 618385-01-6 |
|  | Camostat Mesilate (CM) | C_21_H_26_N_4_O_8_S | 59721-29-8 |
| Natural Bioactive Compound | Vaccarin | C_32_H_38_O_19_ | 53452-16-7 |
|  | Ganoderic acid | C_30_H_44_O_7_ | 81907-62-2 |
|  | Curcumin | C_21_H_20_O_6_ | 458-37-7 |
|  | Apocynin | C_9_H_10_O_3_ | 498-02-2 |
|  | Tanshinone IIA | C_19_H_18_O_3_ | 568-72-9 |
|  | Quercetin | C_15_H_10_O_7_ | 117-39-5 |
|  | Astragaloside IV | C_41_H_68_O_14_ | 84687-43-4 |
|  | Patchouli alcohol (PA) | C_15_H_26_O | 5986-55-0 |
|  | Osthole | C_15_H_16_O_3_ | 484-12-8 |
|  | Pinocembrin (PIN) | C_15_H_12_O_4_ | 68745-38-0 |
|  | Alpha-mangostin (α-MG) | C_24_H_26_O_6_ | 6147-11-1 |
|  | Fraxetin | C_10_H_8_O_5_ | 574-84-5 |
|  | Apigenin | C_15_H_10_O_5_ | 520-36-5 |
|  | Guaiacol | C_7_H_8_O_2_ | 90-05-1 |
|  | Vanillin | C_8_H_8_O_3_ | 121-33-5 |
|  | Puerarin | C_21_H_20_O_9_ | 3681-99-0 |
|  | CS-N | Mixture (Cordyceps sinensis nucleoside/nucleotide-enriched fraction, containing 10 components including guanosine, uridine, and adenosine) | N/A |
|  | S-Methylmethionine (SMM) | C_6_H_14_NO_2_S | 6708-35-6 |
|  | Higenamine | C_16_H_17_NO_3_ | 5843-65-2 |
|  | Resveratrol | C_14_H_12_O_3_ | 501-36-0 |
|  | Cardamonin (CAD) | C_16_H_14_O_4_ | 19309-14-9 |
|  | trans-2,3,5,4'-tetrahydroxystilbene 2-O-β-d-glucopyranoside | C_20_H_22_O_9_ | 82373-94-2 |
|  | Nimbidiol | C_17_H_22_O_3_ | 113332-25-5 |
|  | Melittin | C_131_H_229_N_39_O_31_ | 37231-28-0 |
|  | Ginsenoside Rg1 | C_42_H_72_O_14_ | 22427-39-0 |
|  | Allicin | C_6_H_10_OS_2_ | 539-86-6 |
